# Direct-acting oral anticoagulants (DOACs) in pregnancy: new insight from VigiBase^®^

**DOI:** 10.1038/s41598-019-43715-4

**Published:** 2019-05-10

**Authors:** Maurizio Sessa, Annamaria Mascolo, Torbjörn Callréus, Annalisa Capuano, Francesco Rossi, Morten Andersen

**Affiliations:** 1 Department of Drug Design and Pharmacology, University of Copenhagen, Jagtvej 160, 2100 København Ø, Denmark; 2 Campania Pharmacovigilance and Pharmacoepidemiology Regional Centre, Department of Experimental Medicine, University of Campania “L. Vanvitelli”, Via Santa Maria di Costantinopoli 16, 80138 Naples, Italy

**Keywords:** Interventional cardiology, Epidemiology

## Abstract

We aimed to perform an analysis of individual case safety reports retrieved after the Standardized MedDRA Query “Pregnancy and neonatal topics” for which Direct-Acting Oral Anticoagulants (DOACs) were claimed as suspected/interacting drugs. Additionally, to investigate if exists a disproportion of cases reporting “Pregnancy and neonatal topics” adverse events rather than other adverse events for DOACs in comparison with all other drugs registered in VigiBase or warfarin. VigiBase, the World Health Organization (WHO)’s global database of individual case safety reports was used as data source. Forty-two cases of abortion were detected of which 18 (42.8%) had alternative causes for its occurrence. Fourteen cases reported congenital anomaly (8 cases) or low birth weight baby/fetal growth restriction (6 cases) of which 62.5% and 33.3% had at least one confounder, respectively. In the disproportionality analyses, a potential safety signal for spontaneous abortion emerged for rivaroxaban (Reporting Odds Ratio, *ROR* 2.70; 95% CI 1.79–4.07) and apixaban (ROR 6.76; 95% CI 2.99–15.25). However, when the same analyses were performed using only cases without alternative causes, no statistically significant associations for rivaroxaban when compared to all other drugs (ROR 1.05; 95% CI 0.54–2.02) or warfarin (ROR 0.79; 95% CI 0.47–1.32) were found. For apixaban, we found a statistically significant ROR for induced abortion when compared to all other drugs or warfarin. For the majority of cases claiming DOACs-induced teratogenic effects, spontaneous or induced abortion there was at least one alternative cause explaining the occurrence of the adverse events. For rivaroxaban, when cases without confounders were considered, no safety signals emerged. However, for apixaban, we found a potential safety signal suggesting an increased probability of reporting spontaneous/induced abortion rather than other events when compared to all other drugs or warfarin.

## Introduction

Currently, low molecular weight heparins represent the gold standard for the prophylaxis of venous thromboembolism during pregnancy. In selected clinical scenarios and timeframe of pregnancy, unfractionated heparin and low dose vitamin K antagonist may represent therapeutic alternatives. Direct-Acting Oral Anticoagulants (DOACs) are not recommended in pregnancy^1,2^. However, it was recently questioned in the scientific literature if DOACs may represent a valuable drug class in the therapeutic armamentarium of thrombosis’ prophylaxis in pregnancy^3^. From a clinical perspective, the main reasoning for discussing their use in pregnancy is based on the observation that DOACs may have intrinsic advantages for pregnant women when compared to vitamin K antagonists, such as the rapid withdrawal in case of premature delivery and a shortened antepartum interruption period, due to their shorter half-life^3,4^. Additionally, from a drug safety perspective, recent evidence suggests they may not have the same magnitude of embryotoxicity as the vitamin K antagonists^5^. In this regard, it should be highlighted that despite promising, clinical evidence on the benefit/risk associated with DOACs for the mother and fetus is scarce and needed^1,2^. To date, no clinical trials have been conducted for DOACs in pregnancy and current concerns are based on the observation that DOACs can cross the placenta/reach the fetus and have the potential to cause reproductive toxicity^2^. No study has been conducted on available cases of DOACs-induced teratogenesis in order to investigate the role of comorbidities, concurrent teratogenic drugs (whenever reported), temporal and biological plausibility of drug-event couples, i.e. the presence of confounders that could potentially explain the outcome and/or reduce the strength of the individual causality between the drug and the adverse event^6^. Moreover, a disproportionality to quantify if, for DOACs, the probability of reporting teratogenic event rather than any other event is higher compared to the probability for all other drugs or for other oral anticoagulants drugs currently used in pregnancy is lacking. Considering aforementioned gaps in knowledge, we aimed to perform a case series of all individual case safety reports enlisting adverse events included in the Standardized Medical Dictionary for Regulatory Activities (MedDRA) Query (SMQ) “Pregnancy and neonatal topics” for which Direct-Acting Oral Anticoagulants (DOACs) were claimed as suspected/interacting drugs. Additionally, to investigate if exists a disproportion of cases reporting “Pregnancy and neonatal topics” adverse events rather than other adverse events for DOACs in comparison with all other drugs registered in VigiBase or warfarin.

## Methods

### Data source

In this study, we used VigiBase as the data source. VigiBase is the largest database of individual case safety records in the world with more than 16 million individual case reports of suspected adverse drug reaction of medicine sent by 131 countries participating to the World Health Organization (WHO) Programme for International Drug Monitoring^7^. VigiBase virtually covers the global population with a high standard of coherence and quality of the data. VigiBase use standardized dictionaries to code adverse events (WHO Adverse Reactions Terminology/MedDRA, and WHO International Statistical Classification of Diseases) and medicinal products (WHODrug) to guarantee effective and accurate data management and analysis. For each individual case safety report, we retrieved demographic, clinical and administrative characteristics/information of the case, ongoing and past pharmacological treatment (including dosage regimen, route of administration, therapy start/end dates, the indication of use, dechallenge and rechallenge). Additionally, it is possible to retrieve information on comorbidities, adverse events (coded according to the medical dictionary for regulatory activities), the source of the reports and the professional qualification of the reporter, and adverse event’s outcomes.

### Data extraction strategy

The Uppsala Monitoring Centre that developed and maintains VigiBase on behalf of the WHO was requested to extract all individual case reports recorded in the VigiBase from 01/01/1967 to 13/07/2017 (date format: DDMMYYYY) having DOACs as suspected/interacting drugs for which there was reported at least one adverse event included in the MedDRA SMQ “Pregnancy and neonatal topics”. With this research strategy, we obtained a dataset that included 764 individual case safety reports. From this preliminary dataset, we extracted our final dataset which was composed of individual case safety reports claiming DOACs-induced teratogenic events or abortion for which DOACs had a temporal and biological plausible correlation with the events (for the formal definition of temporal and biological plausibility please refer to section 2.3). The same procedure was performed also for individual case safety report having warfarin as suspected/interacting drug in order to perform symmetric disproportionality sensitivity analyses as described in section 2.4.

To perform disproportionality analyses, we also requested aggregated data for all drugs included in the anatomical therapeutic chemical classification (ATC) code B01A and for all other drugs. In particular, we requested the number of individual case safety reports having: (1) preferred terms enlisted in the MedDRA SMQ “Pregnancy and neonatal topics”; (2) all other preferred terms.

Considering the drug-event pairs under investigation, for disproportionality analyses, we used the female gender and the age of cases (the range between 14 and 40 years) as filters (Supplementary Figs 1–3), i.e. we restricted the disproportionality analyses to the population of cases of female gender in the fertile age. This restriction was performed because we believed it necessary in relation to pregnancy-related outcomes for a meaningful computation of the Reporting Odds Ratio (ROR), or rather the statistical measure used in the disproportionality analyses^8^. In such evaluations, the inclusion of adverse events occurred in male cases or in women that were not in fertile age may lead to bias. By using the aforementioned filters, we restricted two elements of the ROR, the “other adverse events for the drug of interest” and “other adverse events for all the other drugs”.

A sensitivity analysis was performed by restricting our data source to the period 2009–2017.

### Case by case assessment

For each individual case safety report, a team composed of clinical pharmacologists and a cardiologist with long-time experience in the Pharmacovigilance field assessed the temporal and biological plausibility for each drug event couple as well as the role of comorbidities and concurrent drugs on the reported adverse events, including potential drug-drug interactions. The case-by-case assessment was composed of three steps.

In the first step, we evaluated the temporal and biological plausibility for all drug-event pairs by looking at the clinical and demographic characteristics of cases, the treatment period and the time to the event. In particular, we considered a drug as being used during pregnancy if, in the individual case report, it was codified as one of the following preferred terms: (1) drug exposure during pregnancy; (2) drug exposure in pregnancy; (3) exposure during pregnancy; (4) fetal exposure during pregnancy; (5) maternal exposure during pregnancy; (6) fetal exposure during pregnancy, first trimester; (7) drug exposure in utero; (8) maternal exposure during pregnancy, first trimester; (9) fetal exposure during pregnancy, first trimester and (10) drug exposure via mother. Additionally, we considered drugs as used in pregnancy if the route of administration was flagged as transplacental i.e. the fetus was exposed to DOACs through the mother. We considered a drug-event couple as temporal or biological implausible if the exposure period was incompatible with the reported event (i.e. 1-day exposure to oxytocin for inducing labor was not considered as biologically plausible for the occurrence of a congenital anomaly). Additionally, we excluded those cases for which the start date of DOACs was subsequent to the date of the adverse event.

In the second step, we assessed the role of comorbidities on adverse events of interest by using both medical judgment and documental approaches. In particular, for the medical judgment approach, the aforementioned team assessed each case in order to identify comorbidities potentially associated with the development of the adverse event. For the documental approach, we screened the scientific literature (MEDLINE) in order to assess if the aforementioned comorbidities were found associated with the occurrence of the reported adverse event.

Finally, in the third step, for each drug, we assessed potential drug-drug interactions that could lead to a pregnancy-related outcome and the teratogenic profile. To identify potential drug-drug interactions, we preliminarily assessed, for each drug, if there was an overlapping period of exposure with any other drug enlisted in the individual case safety report. This procedure was performed by looking at the date of commencement and the date of discontinuation of drugs. For those drugs having an overlapping period of exposure, we assessed the presence of drug-drug interaction by using Micromedex® a software that provides instant access to drug-drug, drug-food, drug-ethanol, and drug-lab test reactions^9^. To assess the teratogenic profile of drugs, we used the new FDA Pregnancy Categories list^10^.

### Case series and disproportionality analyses

All cases retrieved by using the strategy described in section 2.3 were grouped and presented as a case series. For all adverse events listed in the case series that involved DOACs, we performed a disproportionality analysis by using the ROR that was calculated as following using the statistical notation: (Pr (adverse event of interest|drug of interest)/Pr (other adverse events|drug of interest))/(Pr (adverse event of interest|all other drugs or an active comparator)/Pr (other adverse events|all other drugs or an active comparator)) where Pr stands for the reporting probability^8^. According to the European Medicines Agency recommendations, the threshold for signal detection was defined as a ROR lower boundary 95% Confidence Interval ≥1 and a number of cases ≥3^11,12^.

Additionally, we performed a case/non-case analysis i.e. a disproportionality analysis in which selected drugs are used as comparators. We used warfarin as the comparator as it has a spectrum of indication of use that overlaps with DOACs, is a well-known teratogen^13^ and, as DOACs, in the Summary of Product Characteristics (SmPC) is currently not recommended in pregnancy. For the case/non-case analysis, we used the threshold previously described for the disproportionality analysis.

For both disproportionality analyses, sensitivity analyses were performed by using only cases without reported confounders.

Data management was performed using SAS statistical software (version 9.4, SAS Institute Inc., Cary, North Carolina) and analysis was performed using R (version 3.2.2, R Development Core Team).

### Data protection

Data contained in VigiBase are anonymized and it is not possible to access patients’ or reporters’ personal information. The data applicant adhered to all applicable legislation such as, but not limited to, EU and national legislation regarding the protection of personal data (e.g. the Data Protection Directive 95/46/EC and Regulation (EC) No 45/2001, as applicable). The person responsible for the data request, management, and analysis was Dr. Maurizio Sessa.

## Results

**Figure 1:**
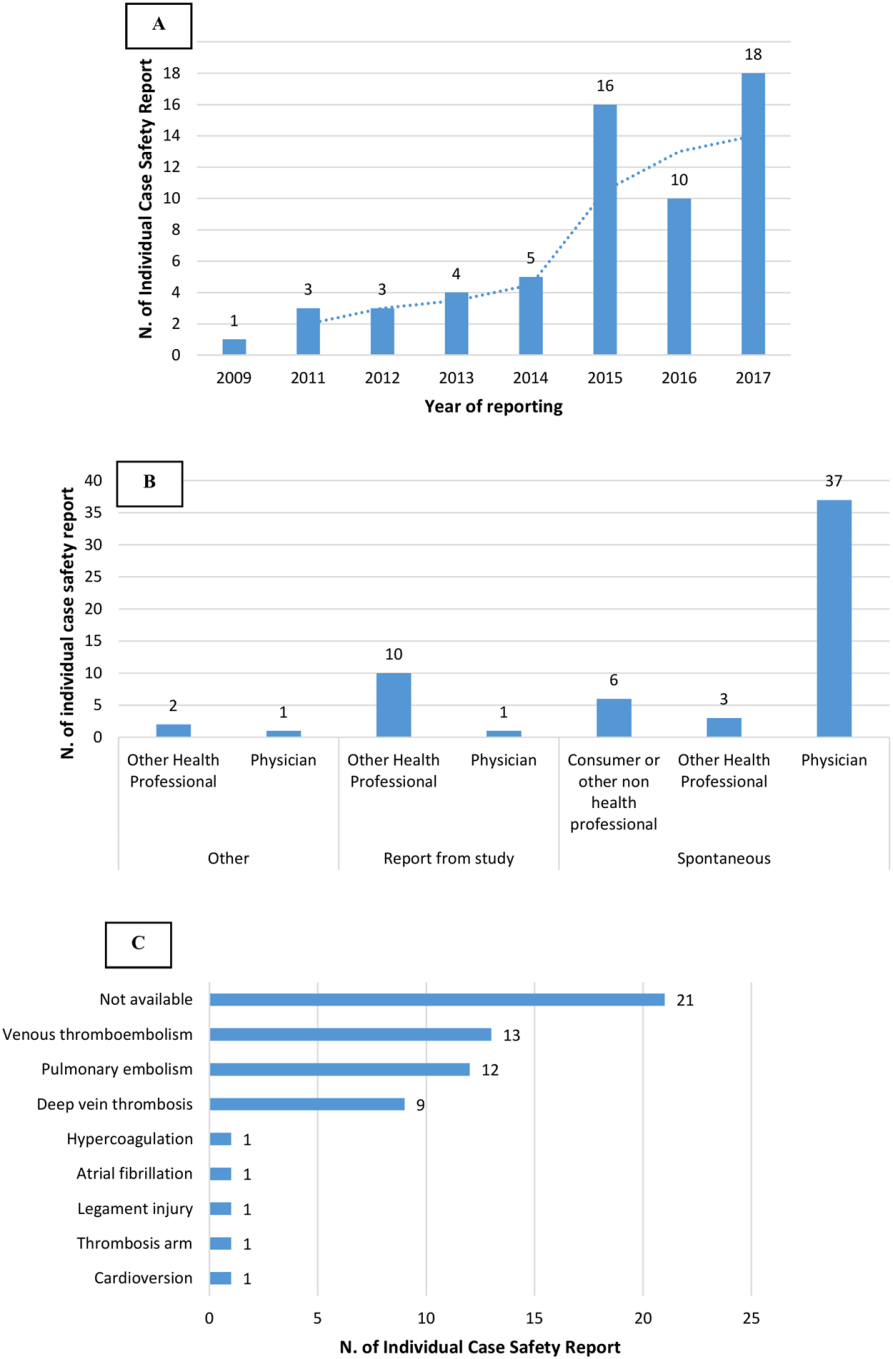
(**A**) Trend over time of selected cases (year of reporting); (**B**) Source and reporter qualification; (**C**) Indication of use of direct-acting oral anticoagulants in selected cases.

In the study period, 60 (7.8%) out of 764 cases fulfilled the data extraction criteria. Excluded cases along with the reasons for exclusion provided in Supplementary Table 1. We observed an incremental trend over years of cases reporting adverse event included in the MedDRA SMQ “Pregnancy and neonatal topics” (Fig. 1A). The majority of retrieved cases derived from Germany (N = 35; 58.3%), while a lower frequency of reports was observed for United Kingdom (N = 5; 8.3%), United States (N = 4; 6.7%), France (N = 3; 5.0%), Switzerland, Sweden, Austria, Hungary (for each N = 2; 3.3%), Norway, Portugal, Ireland, Turkey, and New Zealand (for each N = 1; 1.7%). In all, 46 (76.7%) out of 60 cases were reported through spontaneous reporting systems. Overall, 39 (65.0%) out of 60 cases were medically confirmed or rather reported by physicians, 15 (25.0%) were sent by other healthcare professionals, and only six (10.0%) reports were sent by consumers (Fig. 1B). DOACs were prescribed for more than 21.7% of cases for venous thromboembolism (13 out of 60 cases; 21.7%) or pulmonary embolism (12 out 60 cases; 20.0%) (Fig. 1C).

### Individual case safety report listing adverse event included in the MedDRA SMQ “Pregnancy and neonatal topics”

The characteristics of each case are provided in Supplementary Table 2. In 26 cases (43.3%) we identified an alternative cause for the adverse events. Forty-two cases of abortion were identified of which 18 (42.8%) had an alternative cause for its occurrence. Fourteen cases reported congenital anomaly (8 cases) or low birth weight baby/fetal growth restriction (6 cases) of which, 62.5% and 33.3% had an alternative cause, respectively. Detected alternative causes included maternal age (11 cases; 18.3%) and the co-exposure to drugs (18 cases; 30.0%) or comorbidities (3 cases; 5.0%) having a potential teratogenic effect (in each individual case safety report, more than one confounder can be present) (Table 1, Supplementary Table 2). In 14 (23.3%) cases, we detected major/moderate potential drug-drug interactions (Supplementary Table 2). Raw data from VigiBase are provided in Supplementary Table 3. The case series of individual case safety report having warfarin as the suspected/interacting drug is provided in Supplementary Table 4.

**Table 1:** Adverse events included in the MedDRA Query “Pregnancy and neonatal topics” that were identified during the case-by-case assessment of individual case safety reports.

DOAC	Number of cases without alternative causes for the adverse event	Number of cases with alternative causes for the adverse event	Total*
**Apixaban**			
**Induced abortion**	**5**	**0**	**5**
Pregnancy termination	1	0	1
Spina bifida	0	1	1
Dysmorphism	0	1	1
Face malformation NOS	0	1	1
Unspecified congenital anomaly of heart	0	1	1
Congenital hydronephrosis	0	1	1
**Spontaneous abortion/miscarriage**	**6**	**0**	**6**
Fetal growth retardation	0	1	1
Skeletal dysplasia	0	1	1
Multiple epiphyseal dysplasia	0	1	1
Fetal growth restriction	0	1	1
Neonatal respiratory distress syndrome	0	1	1
Skeletal malformation	0	1	1
Congenital musculoskeletal anomaly	0	1	1
Premature baby	0	1	1
Chondrodystrophy	0	1	1
Brachydactyly	0	1	1
Uterus evacuation	1	0	1
Abortion	1	0	1
Abortion early	1	0	1
Low birth weight baby	1	0	1
**Dabigatran**			
Miscarriage	0	1	1
Induced abortion	1	0	1
Edoxaban			
Spontaneous abortion	1	0	1
**Rivaroxaban**			
**Intrauterine growth retardation/Low birth weight baby**	**4**	**1**	**5**
**Spontaneous abortion/miscarriage**	**9**	**14**	**23**
Abortion	1	0	1
Fetal heartbeat absent	1	0	1
Congenital limb anomaly	1	0	1
Fetal death	1	0	1
Fetal heart rate deceleration	1	0	1
Missed abortion	0	1	1
Facial dysmorphism	1	0	1
Oligohydramnios	0	1	1
Ventricular hypoplasia	0	1	1
Abortion (unspecified)	0	1	1
Congenital cerebral cyst	1	0	1
Congenital anomaly NOS	0	1	1
Congenital cardiac septal defect	0	1	1
Heart disease congenital	0	1	1
Congenital cardiovascular anomaly	0	1	1
Abortion early	0	1	1
Post-abortion bleeding	0	1	1
Induced abortion	0	1	1
Premature delivery	1	0	1

*In each Individual Case Safety Report, more than one adverse event could be reported.

### Disproportionality analyses

**Figure 2:**
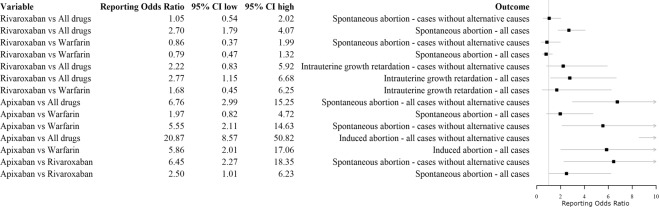
Disproportionality and case/non-case analyses.

**Figure 3:**
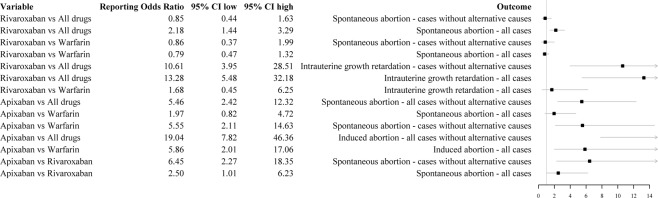
Disproportionality and case/non-case analyses performed in the sensitivity analysis.

In the disproportionality analysis, when cases with and without alternative causes for the adverse event were considered, rivaroxaban (ROR 2.70; 95% CI 1.79–4.07) and apixaban (ROR 6.76; 95% CI 2.99–15.25) had an increased probability of reporting spontaneous abortion compared to other adverse events in comparison to all other drugs. However, when the analysis was restricted to cases without confounders, rivaroxaban did not show a statistically significant estimation (ROR 1.05; 95% CI 0.54–2.02). Similarly, when all cases were used, rivaroxaban did not show a statistically significant ROR for spontaneous abortion in comparison to warfarin (ROR 0.79; 95% CI 0.47–1.32). Similar results were observed when the analysis was restricted only to cases without reported alternative causes for spontaneous abortion (ROR 0.86 95% CI 0.37–1.99). For the association apixaban and spontaneous abortion, we observed no statistically significant ROR if compared to warfarin (ROR 1.97; 95% CI 0.82–4.72) and a statistically significant ROR compared to rivaroxaban (ROR 2.59; 95% CI 1.01–6.23). When only cases without confounders were considered, apixaban showed a statistically significant ROR for spontaneous abortion if compared to warfarin (ROR 5.55; 95% CI 2.11–14.63) or rivaroxaban (ROR 6.45; 95% CI 2.27–18.35). For induced abortion, we found an increased probability of reporting this adverse event as opposed to other adverse events for apixaban when compared to both all other drugs and warfarin. Estimations are provided in Fig. 2. Frequencies used to compute reporting odds ratio are provided in Table 2. The results of the sensitivity analysis performed by restricting our data source to the period 2009–2017 were consistent with those obtained in the main analysis and are provided in Fig. 3 and Supplementary Table 5.

**Table 2:** Two by two tables used to compute reporting odds ratios and 95% confidence intervals.

	Cases without alternative causes		All cases	
**Drug**	**Spontaneous abortion/miscarriage**	**Other adverse events**	**Spontaneous abortion/miscarriage**	**Other adverse events**
**Rivaroxaban**	9	1675	23	1661
**Other drugs**	9644	1878495	9644	1878495
**Drug**	**Spontaneous abortion/miscarriage**	**Other adverse events**	**Spontaneous abortion/miscarriage**	**Other adverse events**
**Rivaroxaban**	9	1675	23	1661
**Warfarin**	14	2241	39	2216
**Drug**	**Intrauterine growth retardation/Low birth weight baby**	**Other adverse events**	**Intrauterine growth retardation/Low birth weight baby**	**Other adverse events**
**Rivaroxaban**	4	1680	5	1679
**Other drugs**	2025	1886114	2025	1886114
**Drug**	**Intrauterine growth retardation/Low birth weight baby**	**Other adverse events**	**Intrauterine growth retardation/Low birth weight baby**	**Other adverse events**
**Rivaroxaban**	4	1680	5	1679
**Warfarin**	2*	2253	4	2251
**Drug**	**Spontaneous abortion/miscarriage**	**Other adverse events**	**Spontaneous abortion/miscarriage**	**Other adverse events**
**Apixaban**	6	173	6	173
**Other drugs**	9644	1878495	9644	1878495
**Drug**	**Spontaneous abortion/miscarriage**	**Other adverse events**	**Spontaneous abortion/miscarriage**	**Other adverse events**
**Apixaban**	6	173	6	173
**Warfarin**	14	2241	39	2216
**Drug**	**Induced abortion**	**Other adverse events**	**Induced abortion**	**Other adverse events**
**Apixaban**	5	174	5	174
**Other drugs**	2596	1885543	2596	1885543
**Drug**	**Induced abortion**	**Other adverse events**	**Induced abortion**	**Other adverse events**
**Apixaban**	5	174	5	174
**Warfarin**	2*	2253	11	2244
**Drug**	**Spontaneous abortion/miscarriage**	**Other adverse events**	**Spontaneous abortion/miscarriage**	**Other adverse events**
**Apixaban**	6	173	6	173
**Rivaroxaban**	9	1675	23	1661

*Not assessable because the number of cases was lower than three.

## Discussion

This is the first study showing that for almost half of the cases describing DOACs-induced adverse events enlisted in the MedDRA SMQ “Pregnancy and neonatal topics” the effect is confounded by factors other than DOACs that could potentially explain the adverse event. Additionally, this study provides, for the first time, a quantitative measure of the ROR of the aforementioned adverse events for DOACs in comparison to all other drugs or warfarin. Despite our results have inherent limitations due to the data source, they can potentially open a new discussion in the scientific community in virtue of the recent recommendations of the European Society of Cardiology guidelines on anticoagulation during pregnancy^1^. The aforementioned guideline suggests that to date, no adequate well-controlled studies are available for DOACs. However, they mentioned that the largest case-series available in the scientific literature, the study conducted Beyer-Westendorf and colleagues^5^, seems to suggest a potential teratogenic effect of DOACs, and in particular for rivaroxaban^1^. Similar results were found by Lameijer *et al*.^14^. Our results do not confirm this, but in contrast, suggest that rivaroxaban is not associated with a disproportional reporting of spontaneous abortion. In this regard, it should be considered that we found an alternative cause for spontaneous abortion/miscarriage or induced abortion for most of the cases described in the article of Beyer-Westendorf and colleagues. Additionally, we found that when cases with confounders were removed from the disproportionality analyses there was no statistically significant evidence to suggest an increased probability of reporting spontaneous abortion/miscarriage or induced abortion rather than other adverse events for rivaroxaban, either if compared to warfarin. It should be highlighted that those cases not having confounders, as also described by Beyer-Westendorf and colleagues, occurred during the first trimester of pregnancy. Overall, the risk of early pregnancy loss is 17–22% during the first trimester where miscarriage could occur spontaneously in absence of noxae^15^. Therefore, also in these cases, we may argue that the first trimester might represent a potential confounder for the occurrence of miscarriage. Another aspect for which our study may open new discussion points is the increased probability of reporting spontaneous or induced abortion for apixaban. A recent study has found that apixaban can easily cross the placenta and, at the steady state, fetal concentrations may range from 35 to 90% of the mother’s concentration having potential direct fetal effects^16^. Despite interesting, our results required further investigation. Considered the discrepancy between the results in the current study and those in Beyer-Westendorf and colleagues, it is our opinion that we do not have sufficient clinical evidence on the safety profile of DOACs in pregnancy and, therefore, no clear answer can be provided on their benefit-risk profile. Most of the available evidence was obtained from observational studies or extrapolation from non-pregnant cohorts mostly small-sized^1^ and lacking information on comorbidities and medicine co-exposure prior to or during pregnancy which may have a strong impact in confounding this drug-event association. As wisely recommended in the aforementioned clinical guidelines, recommendations might be provided only when adequate well-controlled studies will provide the necessary evidence potentially in the set of a common data model from an international collaboration.

### Strength and limitations

The use of VigiBase, a database that virtually covers the entire global population, with regard to adverse events report is considered a strength. Because clinical trials on drug safety of DOACs in pregnancy are unethical to perform, evidence on the role of DOACs-induced teratogenesis in humans can be obtained mainly from observational studies or case series of individual case safety reports. In this context, considering the rarity of both exposure and adverse events, spontaneous reporting systems may represent the only data source able to provide new clinical evidence into the role of DOACs-induced teratogenesis. Although not recommended in pregnancy, women in treatment with DOACs might not be aware of pregnancy at a time when organogenesis has already started and the fetus could be unknowingly exposed during the early embryonic period. Therefore, if adverse events occur, whenever they are reported to regulatory agencies, they could be potentially tracked through individual case safety reports. However, our study’s results should be considered in virtue of a set of limitations. The supplied data come from a variety of sources for which the likelihood of a causal relationship may not be the same in all reports. On the other hand, the aforementioned data source is also a limitation. By using data from the spontaneous reporting system, we cannot rule out the effect biases such as differential reporting, underreporting, lack of denominator and irregular information quality. Therefore, interpretations of adverse reaction data, and particularly those based on comparisons between medicinal products, may be misleading. Any use of this information must consider these factors. Additionally, we cannot exclude the existence of differential reporting of aforementioned drug-event pairs given the labeling difference among DOACs and warfarin. For both disproportionality and case/non-case analyses, we restricted our study outcomes to selected adverse events because only for these adverse events, for at least one DOAC (in particular rivaroxaban and apixaban), were reported at least three individual case safety reports in VigiBase (with and/or without alternative causes). It cannot be ruled out that in the disproportionality and case/non-case analyses the exclusion rate due to alternative causes was similar between DOACs and all other drugs. Finally, it should be highlighted that for three cases involving warfarin (1 spontaneous abortion and 2 induced abortions) adverse events occurred in the setting of an ectopic pregnancy.

## Conclusion

This is the first study showing that for most of the cases describing DOACs-induced adverse events enlisted in the MedDRA SMQ “Pregnancy and neonatal topics” the association is confounded by factors other than DOACs. When cases with alternative causes are removed, on a global scale this study found no evidence from spontaneous reporting systems for an increased probability of reporting spontaneous abortion rather than other adverse events for rivaroxaban when it was compared to all other drugs included in VigiBase or warfarin. For apixaban, we observed an increased probability of reporting the aforementioned event or induced abortion when compared to all other drugs, warfarin or rivaroxaban. The current study results do not allow drawing any conclusions that can be directly implemented into clinical practice.

## Supplementary information

Supplementary material

## Data Availability

All relevant data were included in the manuscript.
