# Supplementary material for: Direct-acting oral anticoagulants (DOACs) in pregnancy: new insight from VigiBase®
Source: Sci Rep. 2019 May 10;9:7236. doi: 10.1038/s41598-019-43715-4 (PMC6510783; doi:10.1038/s41598-019-43715-4)
Supplement: Supplementary file 1 — Supplementary material [file 41598_2019_43715_MOESM1_ESM.pdf]

SUPPLEMENTARY MATERIAL

Direct-acting oral anticoagulants (DOACs) in pregnancy: new insight from VigiBase®.

Maurizio Sessa[1,2], Annamaria Mascolo[2], Torbjörn Callréus[1], Annalisa Capuano[2], Francesco Rossi[2], Morten Andersen[1]

(1) Department of Drug Design and Pharmacology, University of Copenhagen, Copenhagen, Denmark

(2) Campania Pharmacovigilance and Pharmacoepidemiology Regional Center, Department of Experimental Medicine, University of Campania "L. Vanvitelli", Naples, Italy

Running heading: Direct-acting oral anticoagulants in pregnancy.

Corresponding author

Maurizio Sessa MPharm, Ph.D.
ORCID: https://orcid.org/0000-0003-0874-4744

Primary address

Department of Drug Design and Pharmacology,
University of Copenhagen,
Jagtvej 160, 2100 København Ø, Denmark
Phone/Fax: [+45] 52755467
E-mail: maurizio.sessa@sund.ku.dk

Secondary address

Campania Pharmacovigilance and Pharmacoepidemiology Regional Centre
Department of Experimental Medicine,
University of Campania "L. Vanvitelli",
Via Santa Maria di Costantinopoli 16, 80138 Naples, Italy.
Phone/Fax: [+39] 0815667652
E-mail: maurizio.sessa@unicampania.it

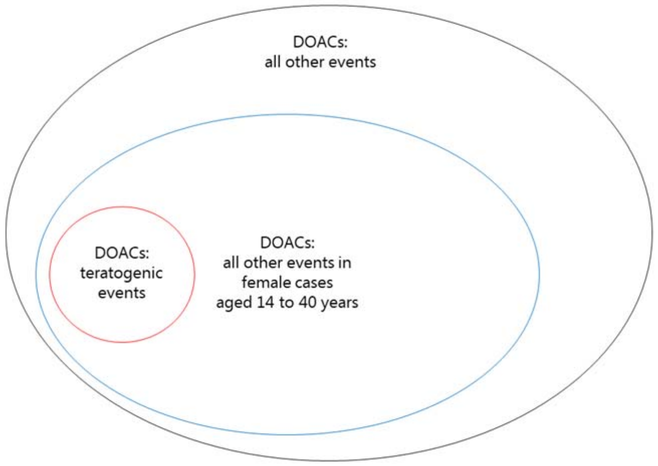

Supplementary figure 1. Data restriction for direct-acting oral anticoagulants (DOACs) in VigiBase®.

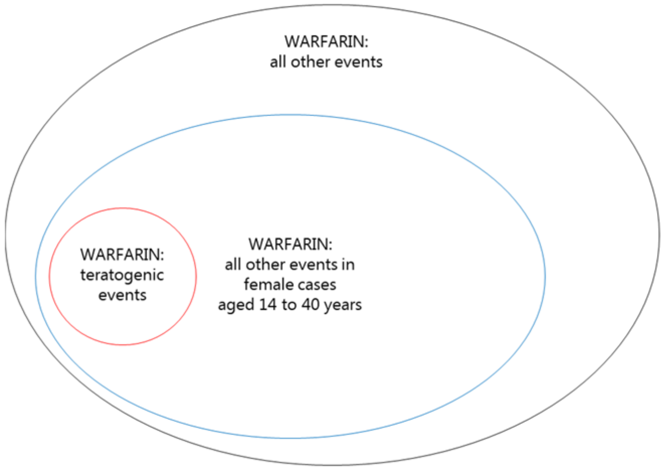

Supplementary figure 2. Data restriction for warfarin in VigiBase®.

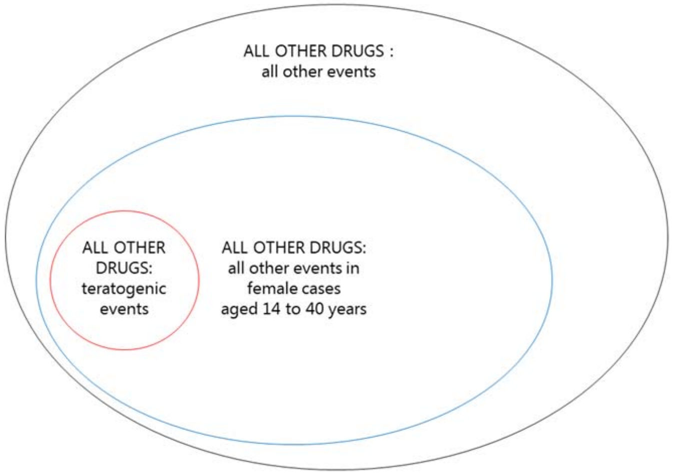

Supplementary figure 3. Data restriction for all other drugs in VigiBase®.

Supplementary table 1. Cases excluded during the case-by-case assessment.

Main cause of exclusion	Number of cases
Hemorrhagic arteriovenous malformation reported as an adverse event in an adult/elder case	404
Arteriovenous malformation reported as an adverse event in an adult/elder case	79
Adverse events didn't' involve the fetus	92
*Near misses	42
Subgaleal hematoma reported as an adverse event in an adult/elder case	26
Hydrocele reported as an adverse event in an adult/elder case	6
Hypertrophic cardiomyopathy reported as an adverse event in an adult/elder case	5
Cephalhaematoma reported as an adverse event in an adult/elder case	4
Hereditary angioedema reported as an adverse event in an adult/elder case	3
Tremor neonatal	3
Atrial sept defect reported as an adverse event in an adult/elder case	2
Aplasia reported as an adverse event in an adult/elder case	2
Factor V Leiden mutation reported as an adverse event in an adult/elder case	2
Hemophilia reported as an adverse event in an adult/elder case	2
Dieulafoy's vascular malformation reported as an adverse event in an adult/elder case	2
Vascular malformation cerebral reported as an adverse event in an adult/elder case	1
Defective color vision reported as an adverse event in an adult/elder case	1
Breast enlargement male unilateral in an adult/elder case	1
Epidermolysis bullosa reported as an adverse event in an adult/elder case	1
Congenital cystic kidney disease reported as an adverse event in an adult/elder case	1
Defective color vision reported as an adverse event in an adult/elder case	1
Left ventricle outflow tract obstruction reported as an adverse event in an adult/elder case	1
Cystic kidney disease reported as an adverse event in an adult/elder case	1
Interatrial foramen secundum defect reported as an adverse event in an adult/elder case	1
Ventricular septal defect reported as an adverse event in an adult/elder case	1
Cleft lip reported as an adverse event in an adult/elder case	1
Chloasma reported as an adverse event in an adult/elder case	1
Gene mutation reported as an adverse event in an adult/elder case	1
Bleeding breast reported as an adverse event in an adult/elder case	1
Vaginal hemorrhage reported as an adverse event in an adult/elder case	1
Cerebral palsy reported as an adverse event in an adult/elder case	1
Left ventricle outflow tract obstruction reported as an adverse event in an adult/elder case	1
Ia septal defect reported as an adverse event in an adult/elder case	1
Epidermolysis reported as an adverse event in an adult/elder case	1
Subarachnoid hemorrhage reported as an adverse event in an adult/elder case	1
Sickle cell anemia reported as an adverse event in an adult/elder case	1
Chromosomal deletion reported as an adverse event in an adult/elder case	1
Huntington's chorea aggravated reported as an adverse event in an adult/elder case	1
Pregnancy ectopic reported as an adverse event in an adult/elder case	1
Phimosis reported as an adverse event in an adult/elder case	1
Cervix hemorrhage uterine reported as an adverse event in an adult/elder case	1
Darier's disease reported as an adverse event in an adult/elder case	1
Coagulopathy reported as an adverse event in an adult/elder case	1
Postpartum in adult/elder hemorrhage	1
Hypertensive cardiomyopathy reported as an adverse event in an adult/elder case	1

Near misses' definition is provided elsewhere (https://www.ema.europa.eu/documents/regulatory-procedural-guideline/good-practice-guide-recording-coding-reporting-assessment-medication-errors_en.pdf).

Supplementary table 2. Case series of individual case safety reports reporting adverse events included in the Standardized MedDRA Query "Pregnancy and neonatal topics" for which Direct-Acting Oral Anticoagulants were claimed as suspected/interacting drugs among those recorded in the VigiBase from 01/01/1967 to 13/07/2017.

**Case' Gender, Age,**<br>**Source of reporting and reporter's qualification**<br>**ADR Seriousness**<br>**Type of report**	**DOAC**<br>**Indication of use**<br>**Dosage Regimen**<br>**Route of administration**	**Case description**	**Confounders**	**Major drug-drug interaction**	**References for confounders**
**Case 1**<br>Female, 0 years old,<br>Spontaneous - Physician<br>Serious – other clinical condition<br>Child-mother report (describing the child)	Rivaroxaban<br>Not available<br>Not available<br>Transplacental	Intrauterine growth retardation and premature delivery	No concurrent comorbidities or co-medications were reported.		
**Case 2**<br>Female, 25 years old,<br>Spontaneous - Physician<br>Serious – other clinical condition<br>Mother-child report (describing the mother)	Dabigatran<br>Cardioversion<br>150 mg per day<br>Oral	Miscarriage and itching	<u>Teratogenic drugs</u><br>*Class N*:<br>1) oxycodone<br>*Class C*:<br>1) pregabalin<br>2) zopiclone<br>3) fluoxetine<br>*Class B*<br>1) tinzaparin<br>2) lansoprazole<br>3) sotalol<br>4) cyclizine	1) sotalol-fluoxetine co-administration can increase the risk of prolonging the QT interval and may result in additive effects and increased risk of ventricular arrhythmias including torsade de pointes and sudden death[17].<br>2) oxycodone-zopiclone co-administration can	[18–24]

				increase the risk of profound sedation, respiratory depression, coma, and death.	
**Case 3**<br>Female, 33 years old,<br>Report from study - Physician<br>Serious – other clinical condition<br>Mother-child report (describing the mother)	Rivaroxaban<br>Pulmonary embolism<br>15 mg per day<br>Oral	Spontaneous abortion	No concurrent comorbidities or co-medications were reported.		
**Case 4**<br>Female, 45 years old,<br>Spontaneous - Consumer<br>Serious – other clinical condition<br>Mother-child report (describing the mother)	Rivaroxaban<br>Thrombosis arm<br>20 mg per day<br>Oral	Spontaneous abortion	Maternal age.		[25]
**Case 5**<br>Female, 23 years old,<br>Spontaneous - Physician<br>Serious – other clinical condition<br>Mother-child report (describing the mother)	Rivaroxaban<br>Venous thromboembolism<br>20 mg per day<br>Oral	Spontaneous abortion	No concurrent comorbidities or co-medications were reported.		
**Case 6**<br>Not available, Not available,<br>Spontaneous - Physician<br>Death<br>Child-mother report (describing the	Rivaroxaban<br>Not available<br>Not available<br>Transplacental	Intrauterine growth retardation	No concurrent comorbidities or co-medications were reported.		

child)					
**Case 7**<br>Female, 37 years old,<br>Spontaneous - Physician<br>Serious – other clinical condition<br>Mother-child report (describing the mother)	Rivaroxaban<br>Venous thromboembolism<br>20 mg per day<br>Oral	Abortion	No concurrent comorbidities or co-medications were reported.		
**Case 8**<br>Female, Not available,<br>Spontaneous - Physician<br>Serious – other clinical condition<br>Mother-child report (describing the mother)	Rivaroxaban<br>Pulmonary embolism<br>Not available<br>Not available	Miscarriage	No concurrent comorbidities or co-medications were reported.		
**Case 9**<br>Female, 33 years old,<br>Spontaneous - Physician<br>Serious – other clinical condition<br>Mother-child report (describing the mother)	Rivaroxaban<br>Venous thromboembolism<br>20 mg per day<br>Not available	Fetal heartbeat absent	No concurrent comorbidities or co-medications were reported.		
**Case 10**<br>Male, Not available,<br>Spontaneous - Physician<br>Not available<br>Child-mother report (describing the	Rivaroxaban<br>Deep vein thrombosis<br>Not available	Intrauterine growth retardation	Teratogenic drugs<br>*Class N*:<br>1) dalteparin<br>*Class C*:	1) rivaroxaban-dalteparin co-administration can increase the risk of bleeding	19

child)	Transplacental		1) betamethasone		
**Case 11**<br>Female, 38 years old,<br>Spontaneous - Physician<br>Serious – other clinical condition<br>Mother-child report (describing the mother)	Rivaroxaban<br>Deep vein thrombosis<br>Not available<br>Transplacental	Miscarriage	Teratogenic drugs<br>*Class N*:<br>1) dalteparin<br><br>Maternal age.	1) rivaroxaban-dalteparin co-administration can increase the risk of bleeding	[19,25]
**Case 12**<br>Female, 32 years old,<br>Spontaneous – Other health professional<br>Caused/Prolonged Hospitalization<br>Mother-child report (describing the mother)	Rivaroxaban<br>Pulmonary embolism<br>15 mg per day<br>Oral	Spontaneous abortion, lack of drug effect, deep vein thrombosis leg and pulmonary embolism	Teratogenic drugs<br>*Class N*:<br>1) warfarin		[19]
**Case 13**<br>Female, 37 years old,<br>Spontaneous – Physician<br>Serious – other clinical condition<br>Mother-child report (describing the mother)	Rivaroxaban<br>Ligament injury<br>10 mg per day<br>Oral	Hemorrhage in early pregnancy and spontaneous abortion	Maternal age.		[25]
**Case 14**<br>Female, 40 years old,<br>Spontaneous – Physician<br>Serious – other clinical condition	Rivaroxaban<br>Venous thromboembolism	Spontaneous abortion	Maternal age.		[25]

Mother-child report (describing the mother)	20 mg per day<br>Oral				
**Case 15**<br>Female, Not available,<br>Report from study – Other healthcare professional<br>Serious – other clinical condition<br>Mother-child report (describing the mother)	Rivaroxaban<br>Venous thromboembolism<br>Not available<br>Oral	Spontaneous abortion	No concurrent comorbidities or co-medications were reported.		
**Case 16**<br>Female, Not available,<br>Report from study – Other healthcare professional<br>Serious – other clinical condition<br>Mother-child report (describing the mother)	Rivaroxaban<br>Venous thromboembolism<br>20 mg per day<br>Oral	Spontaneous abortion	Antiphospholipid syndrome		[26]
**Case 17**<br>Female, Not available,<br>Report from study – Other healthcare professional<br>Serious – other clinical condition<br>Mother-child report (describing the mother)	Rivaroxaban<br>Pulmonary embolism<br>Not available<br>Oral	Spontaneous abortion	Teratogenic drugs<br>*Class N*:<br>1) buprenorphine<br><br>Opioid abuse		[27]

**Case 18**<br>Female, Not available,<br>Report from study – Other healthcare professional<br>Serious – other clinical condition<br>Mother-child report (describing the mother)	Rivaroxaban<br>Venous thromboembolism<br>20 mg per day<br>Oral	Spontaneous abortion	No concurrent comorbidities or co-medications were reported.		
**Case 19**<br>Not available, Not available,<br>Report from study – Other healthcare professional<br>Serious – congenital anomaly<br>Child-mother report (describing the child)	Rivaroxaban<br>Not available<br>Not available<br>Not available	Congenital limb anomaly and fetal death	No concurrent comorbidities or co-medications were reported.		
**Case 20**<br>Female, Not available,<br>Report from study – Other healthcare professional<br>Serious – other clinical condition<br>Mother-child report (describing the mother)	Rivaroxaban<br>Venous thromboembolism<br>10 mg - unknown<br>Not available	Spontaneous abortion	No concurrent comorbidities or co-medications were reported.		
**Case 21**<br>Female, Not available,<br>Report from study – Other healthcare professional<br>Serious – other clinical condition<br>Mother-child report (describing the	Rivaroxaban<br>Pulmonary embolism<br>Not available<br>Not available	Fetal heart rate deceleration	No concurrent comorbidities or co-medications were reported.		

mother)					
**Case 22**<br>Female, 37 years old,<br>Report from study – Other healthcare professional<br>Serious – other clinical condition<br>Mother-child report (describing the mother)	Rivaroxaban<br>Venous thromboembolism<br>10 mg per day<br>Not available	Missed abortion and uterine bleeding	Maternal age.		[25]
**Case 23**<br>Female, 29 years old,<br>Spontaneous – Consumer<br>Serious – other clinical condition<br>Mother-child report (describing the mother)	Rivaroxaban<br>Venous thromboembolism<br>20 mg - unknown<br>Not available	Miscarriage	No concurrent comorbidities or co-medications were reported.		
**Case 24**<br>Female, 37 years old,<br>Spontaneous – Physician<br>Serious – Caused/Prolonged Hospitalization<br>Mother-child report (describing the mother)	Rivaroxaban<br>Pulmonary embolism<br>Not available<br>Oral	Miscarriage	Maternal age.		[25]

**Case 25**<br>Female, 27 years old,<br>Spontaneous – Physician<br>Not available<br>Mother-child report (describing the mother)	Rivaroxaban<br>Pulmonary embolism<br>20 mg - unknown<br>Not available	Miscarriage	Teratogenic drugs<br>*Class N*:<br>1) dalteparin	1) rivaroxaban-dalteparin co-administration can increase the risk of bleeding	19
**Case 26**<br>Female, 27 years old,<br>Spontaneous – Physician<br>Serious – Caused/Prolonged Hospitalization<br>Mother-child report (describing the mother)	Rivaroxaban<br>Pulmonary embolism<br>Not available<br>Oral	Miscarriage	No concurrent comorbidities or co-medications were reported.		
**Case 27**<br>Not available, Not available,<br>Other – Other healthcare professional<br>Serious – other clinical condition<br>Child-mother report (describing the child)	Rivaroxaban<br>Not available<br>Not available<br>Transplacental	Renal pelvis dilatation and facial dysmorphism	No concurrent comorbidities or co-medications were reported.		
**Case 28**<br>Female, 27 years old,<br>Spontaneous – Physician<br>Serious – Caused/Prolonged Hospitalization<br>Mother-child report (describing the mother)	Apixaban<br>Not available<br>Not available<br>Not available	Induced abortion	No concurrent comorbidities or co-medications were reported.		

**Case 29**<br>Female, Not available,<br>Other – other healthcare professional<br>Serious – other clinical condition<br>Child-mother report (describing the child)	Rivaroxaban<br>Not available<br>Not available<br>Transplacental	Low birth weight baby	No concurrent comorbidities or co-medications were reported.		
**Case 30**<br>Female, 30 years old,<br>Spontaneous – physician<br>Serious – other clinical condition<br>Mother-child report (describing the mother)	Rivaroxaban<br>Venous thromboembolism<br>20 mg per day<br>Not available	Spontaneous abortion	No concurrent comorbidities or co-medications were reported.		
**Case 31**<br>Male, Not available,<br>Spontaneous – physician<br>Serious – congenital anomaly<br>Child-mother report (describing the child)	Rivaroxaban<br>Venous thromboembolism<br>Not available<br>Transplacental	Oligohydramnios and ventricular hypoplasia	<u>Teratogenic drugs</u><br>*Class N*:<br>1) acetylsalicylic acid	1) acetylsalicylic acid – rivaroxaban co-administration can increase the risk of bleeding	[28]
**Case 32**<br>Female, 36 years old,<br>Report from study – other healthcare professional<br>Serious – other clinical condition<br>Mother-child report (describing the mother)	Rivaroxaban<br>Venous thromboembolism<br>20 mg - unknown<br>Not available	Miscarriage	Maternal age.		[25]

**Case 33**<br>Female, 23 years old,<br>Spontaneous – Physician<br>Serious – other clinical condition<br>Mother-child report (describing the mother)	Apixaban<br>Deep venous thrombosis<br>5 mg per day<br>Oral	Pregnancy termination	Teratogenic drugs<br>*Class B*:<br>1) enoxaparin	1) enoxaparin-apixaban co-administration can increase the risk of bleeding	19
**Case 34**<br>Female, 34 years old,<br>Spontaneous – Physician<br>Serious – other clinical condition<br>Mother-child report (describing the mother)	Rivaroxaban<br>Deep vein thrombosis<br>20 mg per day<br>Not available	Abortion (unspecified)	Maternal age.		25
**Case 35**<br>Not available, Not available,<br>Spontaneous – Physician<br>Serious – congenital anomaly<br>Child-mother report (describing the child)	Apixaban<br>Pulmonary embolism<br>Not available<br>Transplacental	Spina bifida, dysmorphism, face malformation NOS, unspecified congenital anomaly of heart, and congenital hydronephrosis	Teratogenic drugs<br>*Class N*:<br>1) oxazepam<br>*Class D*:<br>1) desogestrel<br>*Class C*:<br>1) zopiclone	1) oxazepam – zopiclone co-administration can increase the risk of central nervous system- and/or respiratory-depressant effects.	19
**Case 36**<br>Male, Not available,<br>Spontaneous – Consumer<br>Serious – congenital anomaly<br>Child-mother report (describing the child)	Rivaroxaban<br>Not available<br>10 mg per day<br>Transplacental	Congenital cerebral cyst	No concurrent comorbidities or co-medications were reported.		

**Case 37**<br>Not available, Not available,<br>Spontaneous – Physician<br>Serious – other clinical condition<br>Child-mother report (describing the child)	Rivaroxaban<br>Deep venous thrombosis prophylaxis<br>10 mg per day<br>Transplacental	Spontaneous abortion	<u>Teratogenic drugs</u><br>*Class B*:<br>1) enoxaparin	1) enoxaparin-apixaban co-administration can increase the risk of bleeding	[19]
**Case 38**<br>Male, Not available,<br>Spontaneous – Consumer<br>Not available<br>Child-mother report (describing the child)	Rivaroxaban<br>Not available<br>Not available<br>Intra-uterine	Congenital anomaly NOS	<u>Teratogenic drugs</u><br>*Class A*:<br>1) levothyroxine		[19]
**Case 39**<br>Female, Not available,<br>Spontaneous – Physician<br>Serious – other clinical condition<br>Mother-child report (describing the mother)	Apixaban<br>Not available<br>Not available<br>Not available	Miscarriage	No concurrent comorbidities or co-medications were reported.		
**Case 40**<br>Female, Not available,<br>Spontaneous – Physician<br>Serious – other clinical condition<br>Mother-child report (describing the mother)	Apixaban<br>Not available<br>Not available<br>Not available	Induced abortion	No concurrent comorbidities or co-medications were reported.		

**Case 41**<br>Female, Not available,<br>Spontaneous – Physician<br>Serious – other clinical condition<br>Mother-child report (describing the mother)	Apixaban<br>Not available<br>Not available<br>Not available	Miscarriage	No concurrent comorbidities or co-medications were reported.		
**Case 42**<br>Female, Not available,<br>Spontaneous – Physician<br>Serious – other clinical condition<br>Mother-child report (describing the mother)	Apixaban<br>Not available<br>Not available<br>Not available	Induced abortion	No concurrent comorbidities or co-medications were reported.		
**Case 43**<br>Female, Not available,<br>Spontaneous – Physician<br>Serious – other clinical condition<br>Mother-child report (describing the mother)	Apixaban<br>Not available<br>Not available<br>Not available	Miscarriage	No concurrent comorbidities or co-medications were reported.		
**Case 44**<br>Female, Not available,<br>Spontaneous – Physician<br>Serious – other clinical condition<br>Mother-child report (describing the mother)	Apixaban<br>Not available<br>Not available<br>Not available	Induced abortion	No concurrent comorbidities or co-medications were reported.		

**Case 45**<br>Female, Not available,<br>Spontaneous – Physician<br>Serious – other clinical condition<br>Mother-child report (describing the mother)	Apixaban<br>Not available<br>Not available<br>Not available	Induced abortion	No concurrent comorbidities or co-medications were reported.		
**Case 46**<br>Female, Not available,<br>Report from study – Other healthcare professional<br>Serious – other clinical condition<br>Mother-child report (describing the mother)	Rivaroxaban<br>Not available<br>20 mg per day<br>Not available	Miscarriage	No concurrent comorbidities or co-medications were reported.		
**Case 47**<br>Not available, Not available,<br>Other – Physician<br>Serious – death<br>Child-mother report (describing the child)	Rivaroxaban<br>Not available<br>Not available<br>Transplacental	Congenital cardiac septal defect, cardiac disorder, heart disease congenital and congenital cardiovascular anomaly	<u>Teratogenic drugs</u><br>*Class N*:<br>1) aliskiren<br>2) dalteparin<br>3) acetylsalicylic acid<br>4) metamizole<br>5) hydroxychloroquine<br>*Class X*:<br>1) simvastatin<br>*Class D*:<br>1) ramipril<br>*Class C*:<br>1) ezetimibe	1) rivaroxaban – dalteparin co-administration can increase the risk of bleeding.<br>2) ramipril – aliskiren co-administration can increase the risk of adverse events including renal complications, hyperkaliemia, and hypotension.<br>3) acetylsalicylic acid –	[19,29–32]

			2) pantoprazole<br>3) salbutamol<br>4) tacrolimus<br>5) prednisolone<br>*Class B*:<br>1) torsemide	rivaroxaban co-administration can increase the risk of bleeding.<br>4) acetylsalicylic acid – dalteparin co-administration can increase the risk of bleeding.<br>5) acetylsalicylic acid – tacrolimus co-administration can increase the risk severe renal impairment.	
**Case 48**<br>Female, Not available,<br>Spontaneous – Physician<br>Serious – other clinical condition<br>Mother-child report (describing the mother)	Rivaroxaban<br>Deep venous thrombosis<br>20 mg per day<br>Not available	Abortion early	<u>Teratogenic drugs</u><br>*Class A*:<br>1) levothyroxine		[19]
**Case 49**<br>Female, 0 years,<br>Spontaneous – Physician<br>Serious – Caused/Prolonged Hospitalization<br>Child-mother report (describing the child)	Apixaban<br>Not available<br>Not available<br>Transplacental	Flaccidity, fetal growth retardation, skeletal dysplasia, multiple epiphyseal dysplasia, fetal growth restriction, congenital musculoskeletal anomaly, neonatal respiratory distress syndrome, skeletal malformation, cyanosis, bradycardia,	<u>Teratogenic drugs</u><br>*Class N*:<br>1) hydroxychloroquine<br>2) atosiban<br>*Class X*:<br>1) macitentan	1) multiple potential drug-drug interactions	[19,20,33]

		posture abnormal, premature baby, hypotonia, chondrodystrophy, and brachydactyly	*Class D*:<br>1) azathioprine<br>*Class C*:<br>1) treprostinil<br>2) prednisolone<br>3) iloprost<br>*Class B*:<br>1) sildenafil<br>2) tinzaparin		
**Case 50**<br>Female, Not available,<br>Spontaneous – Physician<br>Serious – other clinical condition<br>Mother-child report (describing the mother)	Apixaban<br>Not available<br>Not available<br>Not available	Uterus evacuation and abortion	No concurrent comorbidities or co-medications were reported.		
**Case 51 -**<br>Female, 38 years old,<br>Spontaneous – Physician<br>Serious – other clinical condition<br>Mother-child report (describing the mother)	Rivaroxaban<br>Atrial fibrillation<br>20 mg per day<br>Not available	Spontaneous abortion and post-abortion bleeding	<u>Teratogenic drugs</u><br>*Class N*:<br>1) digitoxin<br>*Class C*:<br>1) metoprolol<br><br>Maternal age	1) metoprolol – digitoxin co-administration can increase the risk of bradycardia.	[19,25]

**Case 52**<br>Male, 0 years old,<br>Spontaneous – Physician<br>Serious – other clinical condition<br>Child-mother report (describing the child)	Rivaroxaban<br>Pulmonary embolism<br>Not available<br>Transplacental	Low birth weight baby	No concurrent comorbidities or co-medications were reported.		
**Case 53**<br>Not available, Not available,<br>Spontaneous – Consumer<br>Serious – other clinical condition<br>Mother-child report (describing the mother)	Apixaban<br>Not available<br>Not available<br>Not available	Low birth weight baby	No concurrent comorbidities or co-medications were reported.		
**Case 54**<br>Female, Not available,<br>Spontaneous – Consumer<br>Serious – other clinical condition<br>Mother-child report (describing the mother)	Edoxaban<br>Pulmonary embolism<br>60 mg per day<br>Oral	Spontaneous abortion and spotting vaginal	No concurrent comorbidities or co-medications were reported.		
**Case 55**<br>Female, 26 years old,<br>Spontaneous – Physician<br>Serious – other clinical condition<br>Mother-child report (describing the mother)	Apixaban<br>Deep vein thrombosis<br>5 mg per day<br>Oral	Spontaneous abortion and abortion early	No concurrent comorbidities or co-medications were reported.		

**Case 56**<br>Female, 26 years old,<br>Spontaneous – Physician<br>Serious – death<br>Mother-child report (describing the mother)	Apixaban<br>Deep vein thrombosis<br>10 mg per day<br>Oral	Spontaneous abortion	No concurrent comorbidities or co-medications were reported.		
**Case 57**<br>Female, 19 years old,<br>Spontaneous – Physician<br>Serious – other clinical condition<br>Mother-child report (describing the mother)	Dabigatran<br>Hypercoagulation<br>Not available<br>Not available	Induced abortion	No concurrent comorbidities or co-medications were reported.		
**Case 58**<br>Female, 36 years old,<br>Spontaneous – Other healthcare professional<br>Serious – other clinical condition<br>Mother-child report (describing the mother)	Rivaroxaban<br>Pulmonary embolism<br>20 mg per day<br>Oral	Spontaneous abortion	<u>Teratogenic drugs</u><br>*Class N*:<br>1) acetylsalicylic acid<br>2) clonazepam<br>*Class C*:<br>1) hydrocodone<br>*Class B*:<br>1) enoxaparin<br><br>Maternal age	1) clonazepam – hydrocodone co-administration can increase the risk of respiratory distress, coma, and even death.<br>2) acetylsalicylic acid – enoxaparin co-administration can increase the risk of bleeding.<br>3) enoxaparin – rivaroxaban co-administration can	[19,25,33]

				increase the risk of bleeding.<br>4) acetylsalicylic acid – rivaroxaban co-administration can increase the risk of bleeding.	
**Case 59**<br>Female, 35 years old,<br>Spontaneous – Other healthcare professional<br>Serious – other clinical condition<br>Mother-child report (describing the mother)	Rivaroxaban<br>Not available<br>Not available<br>Oral	Induced abortion	Teratogenic drugs<br>*Class N*:<br>1) clonazepam<br>*Class D*:<br>1) mercaptopurine<br>*Class C*:<br>1) prednisone<br>*Class B*:<br>1) cyclobenzaprine<br>2) prednisone<br><br>Maternal age<br>Crohn's disease	1) mercaptopurine – certolizumab co-administration can increase the risk of serious and potentially life-threatening infections.<br>2) prednisone – certolizumab co-administration can increase the risk of serious and potentially life-threatening infections.	[19,25,33]
**Case 60**<br>Female, 33 years old,<br>Spontaneous – Physician<br>Serious – other clinical condition	Rivaroxaban<br>Deep venous thrombosis	Spontaneous abortion	Teratogenic drugs<br>*Class B*:<br>1) enoxaparin	1) enoxaparin – rivaroxaban co-administration can	[19,25]

Mother-child report (describing the mother)	20 mg - unknown<br>Oral			increase the risk of bleeding.	

Supplementary table 3. Raw data from VigiBase (case format provided by the Uppsala Monitoring Centre).

Age/Sex	Suspected (S), interacting (I) or concomitant (C) drugs	Reactions (MedDRA PT)	Time to event	Dechallenge; rechallenge	Outcome
26/F	Apixaban (S)	Abortion early, Maternal exposure during pregnancy, Prescribed underdose	-	Drug withdrawn/Reaction abated ; -	recovered
26/F	Apixaban (S)	Abortion early, Abortion spontaneous, Maternal exposure during pregnancy, Prescribed underdose, Product use issue	6 months, 6 months	Drug withdrawn/-, Drug withdrawn/Reaction abated ; -	recovered, unknown
-/F	Edoxaban (S)	Abortion spontaneous, Maternal exposure during pregnancy, Vaginal hemorrhage	-	Drug withdrawn/-, Drug withdrawn/Reaction abated ; -	recovered, unknown
-/F	Apixaban (S)	Abortion spontaneous, Maternal exposure during pregnancy, Product use issue	-	Dose not changed/-, Dose not changed/Reaction abated ; -	recovered, unknown
-1/M	Rivaroxaban (S)	Fetal exposure during pregnancy, Low birth weight baby	-	-	unknown
-/F	Apixaban (S)	Abortion spontaneous, Maternal exposure during pregnancy, Product use issue	-	-, -/Reaction abated ; -	recovered, unknown
-/F	Apixaban (S)	Abortion induced, Maternal exposure during pregnancy, Product use issue	-	-, -/Reaction abated ; -	recovered, unknown
-/F	Apixaban (S)	Abortion spontaneous, Maternal exposure during pregnancy, Product use issue	-	-, -/Reaction abated ; -	recovered, unknown
-/F	Apixaban (S)	Abortion induced, Maternal exposure during pregnancy, Product use issue	-	-, -/Reaction abated ; -	recovered, unknown
-/F	Apixaban (S)	Abortion spontaneous, Maternal exposure during pregnancy, Product use issue	-	-, -/Reaction abated ; -	recovered, unknown
-/F	Apixaban (S)	Abortion spontaneous, Maternal exposure during pregnancy, Product use issue	-	-, -/Reaction abated ; -	recovered, unknown
-/F	Apixaban (S)	Abortion induced, Maternal exposure during pregnancy, Product use issue	-	-, -/Reaction abated ; -	recovered, unknown
-/M	Rivaroxaban (S) Levothyroxine (C)	Congenital anomaly	-	-/Not applicable ; No rechallenge/Not applicable	unknown
-/F	Apixaban (S)	Abortion, Maternal exposure during pregnancy, Product use issue, Uterine dilation and evacuation	-	-, -/Reaction abated ; -	recovered, unknown
-/-	Enoxaparin, Rivaroxaban (S)	Abortion spontaneous incomplete, Exposure during pregnancy	-22 days	Dose not changed/Reaction abated ; -, Drug withdrawn/Reaction abated ; -	recovered

38/F	Rivaroxaban (S) Digitoxin, Metoprolol (C)	Abortion spontaneous, Maternal exposure during pregnancy, Post abortion hemorrhage	-16 days	-	recovered
23/F	Apixaban, Enoxaparin (S)	Abortion induced, Maternal exposure during pregnancy, Product use issue	5 months	-, -/Reaction abated ; -, Drug withdrawn/-, Drug withdrawn/Reaction abated ; -	recovered, unknown
23/F	Rivaroxaban (S)	Abortion spontaneous, Maternal exposure during pregnancy	-	-	unknown
36/F	Rivaroxaban (S)	Abortion spontaneous, Maternal exposure during pregnancy	-	-	unknown
-/M	Acetylsalicylic acid, Rivaroxaban (S)	Oligohydramnios, Ventricular hypoplasia	3 months	Dose not changed/No effect observed ; -, Not applicable/No effect observed ; -	not recovered
34/F	Rivaroxaban (S)	Abortion, Maternal exposure during pregnancy	-	-	-
-/-	Apixaban (S) Desogestrel, Oxazepam, Zopiclone (C)	Congenital hydronephrosis, Dysmorphism, Foetal exposure during pregnancy, Heart disease congenital, Spina bifida	-	-	unknown
30/F	Rivaroxaban (S)	Abortion spontaneous, Maternal exposure during pregnancy	-	Not applicable/-, Not applicable/Reaction abated ; -	recovered, unknown
33/F	Enoxaparin, Rivaroxaban (S)	Abortion spontaneous, Exposure during pregnancy	-	-, -/Reaction abated ; -, Drug withdrawn/- ; Rechallenge/No recurrence, Drug withdrawn/Reaction abated ; Rechallenge/No recurrence	recovered, unknown
-/F	Rivaroxaban (S)	Foetal exposure during pregnancy, Low birth weight baby	-	-	unknown
0/F	Apixaban, Azathioprine, Hydroxychloroquine, Iloprost, Macitentan, Prednisolone, Sildenafil, Tinzaparin, Treprostinil (S) Atosiban, Betamethasone, Drug name/s under assessment for who-dd (C)	Brachydactyly, Bradycardia, Chondrodystrophy, Congenital musculoskeletal anomaly, Cyanosis, Foetal exposure timing unspecified, Foetal growth restriction, Hypotonia, Multiple epiphyseal dysplasia, Neonatal respiratory distress syndrome, Posture abnormal, Premature baby, Skeletal dysplasia	6 months	-	not recovered, unknown
-/-	Rivaroxaban (S)	Dysmorphism, Foetal exposure during pregnancy, Pyelocaliectasis	-	-	unknown
27/F	Rivaroxaban (S)	Abortion spontaneous	-	Drug withdrawn/Reaction	recovered

				abated ; -	
27/F	Rivaroxaban (S) Dalteparin (C)	Abortion spontaneous	-	Not applicable/Reaction abated ; Rechallenge/-	recovered
-/F	Rivaroxaban (S) Levothyroxine (C)	Abortion early, Exposure during pregnancy	-	-	unknown
35/F	Certolizumab, Rivaroxaban (S) Clonazepam, Cyclobenzaprine, Mercaptopurine, Prednisone (C)	Abortion induced, Exposure during pregnancy	-	-/Reaction abated ; Rechallenge/-	recovered
29/F	Rivaroxaban (S)	Abortion spontaneous, Exposure during pregnancy	-	Drug withdrawn/- ; Rechallenge/-	unknown
36/F	Rivaroxaban (S)	Abortion spontaneous, Metrorrhagia	9 months	Drug withdrawn/Reaction abated ; Rechallenge/-	recovered with sequelae
-/-	Acetylsalicylic acid, Aliskiren, Dalteparin, Ezetimibe, Hydroxychloroquine, Metamizole, Pantoprazole, Prednisolone, Ramipril, Rivaroxaban, Salbutamol, Simvastatin, Tacrolimus, Torasemide (S) Metamizole (C)	Cardiac disorder, Cardiac septal defect, Congenital cardiovascular anomaly, Foetal exposure during pregnancy, Heart disease congenital	-	Not applicable/-, Not applicable/Fatal ; -	fatal, unknown
37/F	Rivaroxaban (S)	Abortion missed, Exposure during pregnancy, Off label use, Uterine hemorrhage	-	Dose not changed/-	unknown
-/F	Rivaroxaban (S)	Exposure during pregnancy, Foetal heart rate deceleration abnormality, Product use issue	-	-	unknown
-/F	Rivaroxaban (S)	Abortion spontaneous, Exposure during pregnancy, Incorrect dose administered, Product use issue	-	Drug withdrawn/-	unknown
-/F	Rivaroxaban (S)	Abortion missed, Abortion spontaneous, Exposure during pregnancy, Off label use	-	Drug withdrawn/-	unknown
-/-	Rivaroxaban (S)	Foetal death, Foetal exposure during pregnancy, Limb malformation	-	-	fatal, unknown
-/F	Rivaroxaban (S) Buprenorphine (C)	Abortion spontaneous, Exposure during pregnancy, Product use issue	-	Drug withdrawn/-	unknown
-/F	Rivaroxaban (S)	Abortion spontaneous, Exposure during pregnancy, Product use issue	-	Drug withdrawn/-	unknown
-/F	Rivaroxaban (S)	Abortion spontaneous, Exposure during	-	Drug withdrawn/-	unknown

		pregnancy, Product use issue			
40/F	Rivaroxaban (S)	Abortion spontaneous, Exposure during pregnancy	5 months, 5 months	-	unknown
38/F	Rivaroxaban (S) Dalteparin (C)	Abortion spontaneous	-	Drug withdrawn/Reaction abated ; Rechallenge/-	recovered
-/M	Rivaroxaban (S) Betamethasone, Dalteparin (C)	Foetal exposure during pregnancy, Foetal growth restriction	0 days, 7 months	Drug withdrawn/-	unknown
32/F	Rivaroxaban (S) Warfarin (C)	Abortion spontaneous, Deep vein thrombosis, Drug ineffective, Exposure during pregnancy, Product use issue, Pulmonary embolism	8 months	Drug withdrawn/-	unknown
35/F	Acetylsalicylic acid, Clonazepam, Enoxaparin, Hydrocodone, Rivaroxaban (S)	Abortion spontaneous, Exposure during pregnancy	-	-/Reaction abated ; -, Drug withdrawn/Reaction abated ; Rechallenge/No recurrence	recovered
-/-	Rivaroxaban (S)	Foetal growth restriction	-	Not applicable/Fatal ; -	fatal
23/F	Rivaroxaban (S)	Abortion spontaneous, Exposure during pregnancy, Product use issue	-	-	unknown
45/F	Rivaroxaban (S)	Abortion spontaneous, Pregnancy	-	Not applicable/Reaction abated ; -	recovered
19/F	Dabigatran (S)	Abortion, Abortion induced	-	-	unknown
33/F	Rivaroxaban (S)	Exposure during pregnancy, Foetal heart rate abnormal	-	-	unknown
27/F	Apixaban (S)	Abortion induced, Maternal exposure during pregnancy, Off label use	-	-, -/Reaction abated ; -	recovered, unknown
-/F	Rivaroxaban (S)	Abortion spontaneous, Exposure during pregnancy	-	-	unknown
25/F	Dabigatran (S) Cyclizine, Fluoxetine, Heparin, Lansoprazole, Oxycodone, Pregabalin, Sotalol, Tinzaparin, Zopiclone (C)	Abortion spontaneous, Pruritus	-	Drug withdrawn/- ; Rechallenge/-	unknown
37/F	Rivaroxaban (S)	Abortion, Exposure during pregnancy, Product use issue	-	Drug withdrawn/-	unknown
-/M	Rivaroxaban (S)	Congenital cerebral cyst, Foetal exposure during pregnancy	-	Not applicable/-	unknown
0/F	Rivaroxaban (S)	Foetal growth restriction, Premature delivery	7 months	Not applicable/- ; Rechallenge/-	unknown

37/F	Rivaroxaban (S)	Abortion spontaneous, Hemorrhage in pregnancy, Maternal exposure during pregnancy	13 days	Not applicable/-	unknown
33/F	Rivaroxaban (S)	Abortion spontaneous	3 months, 4 months	Dose not changed/Reaction abated ; -	recovered

Supplementary table 4. Case series of individual case safety reports reporting adverse events included in the Standardized MedDRA Query “Pregnancy and neonatal topics” for which warfarin was claimed as suspected/interacting drug among those recorded in the VigiBase from 01/01/1967 to 13/07/2017.

**Case’ Gender, Age, Source of reporting and reporter’s qualification**<br>**ADR Seriousness**<br>**Type of report**	**Vitamin K antagonist**<br>**Indication of use**<br>**Dosage Regimen**<br>**Route of administration**	**Case description**	**Confounders**	**Major drug-drug interaction**	**References for confounders**
**Case 1**<br>Female, 30 years old, Spontaneous – Consumer<br>Unknown<br>Mother-child report (describing the mother)	Warfarin<br>Cardioembolic stroke<br>Unknown<br>Unknown	Spontaneous abortion	<u>Teratogenic drugs</u><br>*Class N*:<br>1) nadroparin calcium	1) Nadroparin – warfarin co-administration can increase the risk of bleeding	Nadroparin’s Summary of Product Characteristics (SmPC)
**Case 2**<br>Female, 37 years old, Spontaneous – General practitioner<br>Death<br>Mother-child report (describing the mother)	Warfarin<br>Unknown<br>Unknown<br>Unknown	Spontaneous abortion, bleeding from ears, hemorrhage NOS and death intrauterine	<u>Teratogenic drugs</u><br>*Class C*:<br>1) heparin<br><br>Maternal age	1) heparin – warfarin co-administration can increase the risk of bleeding	Warfarin’s SmPC
**Case 3**<br>Female, unknown, Spontaneous - Pharmacist<br>Serious – other clinical condition<br>Mother-child report	Warfarin<br>Unknown<br>Unknown<br>Unknown	Abortion induced	No concurrent comorbidities or co-medications were reported.		

(describing the mother)					
**Case 4**<br>Female, 23 years old,<br>Spontaneous - Physician<br>Serious – other clinical condition<br>Mother-child report (describing the mother)	Warfarin<br>Unknown<br>Unknown<br>Unknown	Abortion induced	<u>Teratogenic drugs</u><br>*Class X*:<br>1) bosentan	1) bosentan – warfarin co-administration may result in decreased anticoagulant effectiveness	[34]
**Case 5**<br>Female, 23 years old,<br>Spontaneous – Other healthcare professional<br>Serious – other clinical condition<br>Mother-child report (describing the mother)	Warfarin<br>Unknown<br>Unknown<br>Unknown	Abortion induced	<u>Teratogenic drugs</u><br>*Class X*:<br>1) bosentan	1) bosentan – warfarin co-administration may result in decreased anticoagulant effectiveness	[34]
**Case 6**<br>Female, unknown,<br>Spontaneous - unknown<br>Serious – other clinical condition<br>Mother-child report (describing the mother)	Warfarin<br>Unknown<br>Unknown<br>Unknown	Spontaneous abortion	No concurrent comorbidities or co-medications were reported.		

**Case 7**<br>Female, 29 years old,<br>Spontaneous - Other healthcare professional<br>Serious – other clinical condition<br>Mother-child report (describing the mother)	Warfarin<br>Antiplatelet therapy (off-label)<br>Unknown<br>Unknown	Spontaneous abortion, human chorionic gonadotropin decreased and ectopic pregnancy	<u>Teratogenic drugs</u><br>*Class D:*<br>1) mycophenolic acid		
**Case 8**<br>Female, 37 years old,<br>Spontaneous - Consumer<br>Serious – other clinical condition<br>Mother-child report (describing the mother)	Warfarin<br>Heart valve replacement<br>Unknown<br>Unknown	Spontaneous abortion	<u>Teratogenic drugs</u><br>*Class B:*<br>1) enoxaparin<br><br>Maternal age	1) enoxaparin – warfarin co-administration can increase the risk of bleeding	Enoxaparin's SmPC
**Case 9**<br>Female, 32 years old,<br>Spontaneous - Consumer<br>Serious – other clinical condition<br>Mother-child report (describing the mother)	Warfarin<br>Unknown<br>Unknown<br>Unknown	Abortion induced and ectopic pregnancy	<u>Teratogenic drugs</u><br>*Class B:*<br>1) epoprostenol<br><br>Pulmonary hypertension		[35]
**Case 10**<br>Female, 45 years old,<br>Spontaneous - Physician<br>Serious – other clinical condition	Warfarin<br>Phlebitis<br>4 mg daily<br>Oral	Spontaneous abortion	Maternal Age		

Mother-child report<br>(describing the mother)					
**Case 11**<br>Female, 28 years old,<br>Spontaneous –<br>Manufacturer<br>Serious – unknown<br>Mother-child report<br>(describing the mother)	Warfarin<br>Unknown<br>Unknown<br>Unknown	Spontaneous abortion,<br>peripheral ischemia,<br>peripheral embolism,<br>compartment syndrome,<br>and vaginal hemorrhage	<u>Teratogenic drugs</u><br>*Class B:*<br>1) enoxaparin	1) enoxaparin – warfarin<br>co-administration can<br>increase the risk of bleeding	Enoxaparin's<br>SmPC
**Case 12**<br>Female, unknown,<br>Spontaneous – Physician<br>Serious – unknown<br>Mother-child report<br>(describing the mother)	Warfarin<br>Unknown<br>Unknown<br>Unknown	Abortion induced	No concurrent comorbidities or co-medications were reported.		
**Case 13**<br>Female, unknown,<br>Spontaneous – Pharmacist<br>Serious – unknown<br>Mother-child report<br>(describing the mother)	Warfarin<br>Unknown<br>Unknown<br>Unknown	Spontaneous abortion,<br>migraine, and suicidal<br>ideation.	No concurrent comorbidities or co-medications were reported.		
**Case 14**<br>Female, 19 years old,<br>Spontaneous – Consumer<br>Serious – other clinical	Warfarin<br>Pulmonary thrombosis<br>Unknown	Spontaneous abortion	<u>Teratogenic drugs</u><br>*Class B:*<br>1) enoxaparin	1) enoxaparin – warfarin<br>co-administration can<br>increase the risk of bleeding	Enoxaparin's<br>SmPC

condition<br>Mother-child report<br>(describing the mother)	Unknown				
**Case 15**<br>Female, unknown,<br>Spontaneous – Consumer<br>Serious – other clinical condition<br>Mother-child report<br>(describing the mother)	Warfarin<br>Thrombosis prophylaxis Unknown<br>Unknown	Spontaneous abortion	No concurrent comorbidities or co-medications were reported.		
**Case 16**<br>Female, unknown,<br>Spontaneous – Consumer<br>Serious – other clinical condition<br>Mother-child report<br>(describing the mother)	Warfarin<br>Thrombosis<br>1 posologic unit<br>Subcutaneous	Spontaneous abortion	<u>Teratogenic drugs</u><br>*Class B:*<br>1) enoxaparin	Enoxaparin and warfarin were not administrated simultaneously	
**Case 17**<br>Female, 25 years old,<br>Spontaneous – Consumer<br>Serious – other clinical condition<br>Mother-child report<br>(describing the mother)	Warfarin<br>Antiphospholipid syndrome<br>1 mg daily<br>Oral	Spontaneous abortion and glomerulonephritis	<u>Teratogenic drugs</u><br>*Class D:*<br>1) mycophenolic acid<br><br>Antiphospholipid syndrome		[26]

**Case 18**<br>Female, unknown,<br>Spontaneous – Consumer<br>Serious – other clinical condition<br>Child-mother report (describing the child)	Warfarin<br>Unknown<br>Unknown<br>Other (exposure via body fluid)	Spontaneous abortion	No concurrent comorbidities or co-medications were reported.		
**Case 19**<br>Female, 27 years old,<br>Spontaneous – Other healthcare professional<br>Serious – Death<br>Mother-child report (describing the mother)	Warfarin<br>Mitral valve replacement<br>Unknown<br>Oral	Spontaneous abortion, hemorrhage intracranial, cardiac valve replacement complication and fetal death	<u>Teratogenic drugs</u><br>*Class B:*<br>1) enoxaparin<br>*Class C:*<br>1) digoxin	1) enoxaparin – warfarin co-administration can increase the risk of bleeding	Enoxaparin's SmPC
**Case 20**<br>Female, unknown,<br>Spontaneous – Consumer<br>Serious – other clinical condition<br>Child-mother report (describing the child)	Warfarin<br>Unknown<br>Unknown<br>Other (exposure via body fluid)	Spontaneous abortion	No concurrent comorbidities or co-medications were reported.		
**Case 21**<br>Female, 23 years old,<br>Spontaneous – unknown<br>Serious – other clinical	Warfarin<br>Prosthetic cardiac valve thrombosis	Spontaneous abortion and vaginal hemorrhage	<u>Teratogenic drugs</u><br>*Class N:*<br>1) acetylsalicylic acid	1) alteplase – warfarin - acetylsalicylic acid co-administration can increase	[36]

condition<br>Mother-child report<br>(describing the mother)	1 mg daily<br>Oral		*Class C:*<br>1) alteplase	the risk of bleeding	
**Case 22**<br>Female, 28 years old,<br>Spontaneous – Physician<br>Serious –<br>Caused/Prolonged<br>Hospitalization<br>Mother-child report<br>(describing the mother)	Warfarin<br>Deep vein thrombosis<br>Unknown<br>Unknown	Miscarriage, vaginal bleeding, hemorrhage and uterus evacuation	<u>Teratogenic drugs</u><br>*Class C*:<br>1) heparin	1) heparin – warfarin co-administration can increase the risk of bleeding	Warfarin's SmPC
**Case 23**<br>Female, unknown,<br>Report from study –<br>Physician<br>Serious – other clinical<br>condition<br>Mother-child report<br>(describing the mother)	Warfarin<br>Unknown<br>Unknown<br>Unknown	Miscarriage	No concurrent comorbidities or co-medications were reported.		
**Case 24**<br>Female, unknown,<br>Report from study –<br>Physician<br>Serious – other clinical<br>condition	Warfarin<br>Unknown<br>Unknown<br>Unknown	Miscarriage	No concurrent comorbidities or co-medications were reported.		

Mother-child report<br>(describing the mother)					
**Case 25**<br>Female, unknown,<br>Spontaneous – Physician<br>Serious – other clinical condition<br>Mother-child report<br>(describing the mother)	Warfarin<br>Anticoagulant therapy<br>Unknown<br>Unknown	Miscarriage	Teratogenic drugs<br>*Class N:*<br>1) Acetylsalicylic acid	1) warfarin - Acetylsalicylic acid co-administration can increase the risk of bleeding	Warfarin's SmPC
**Case 26**<br>Female, unknown,<br>Spontaneous – Physician<br>Serious – other clinical condition<br>Mother-child report<br>(describing the mother)	Warfarin<br>Anticoagulant therapy<br>Unknown<br>Unknown	Miscarriage and hemorrhage	No concurrent comorbidities or co-medications were reported.		
**Case 27**<br>Female, unknown,<br>Spontaneous – Physician<br>Serious – other clinical condition<br>Mother-child report<br>(describing the mother)	Warfarin<br>Anticoagulant therapy<br>Unknown<br>Unknown	Miscarriage	Teratogenic drugs<br>*Class N:*<br>1) Acetylsalicylic acid	1) warfarin - Acetylsalicylic acid co-administration can increase the risk of bleeding	Warfarin's SmPC

**Case 28**<br>Female, unknown,<br>Spontaneous – Physician<br>Serious – other clinical condition<br>Mother-child report (describing the mother)	Warfarin<br>Anticoagulant therapy<br>Unknown<br>Unknown	Miscarriage and hemorrhage	<u>Teratogenic drugs</u><br>*Class N:*<br>1) Acetylsalicylic acid	1) warfarin - Acetylsalicylic acid co-administration can increase the risk of bleeding	Warfarin's SmPC
**Case 29**<br>Female, unknown,<br>Spontaneous – Physician<br>Serious – other clinical condition<br>Mother-child report (describing the mother)	Warfarin<br>Unknown<br>6 mg<br>Unknown	Miscarriage	No concurrent comorbidities or co-medications were reported.		
**Case 30**<br>Female, unknown,<br>Spontaneous – Physician<br>Serious – other clinical condition<br>Mother-child report (describing the mother)	Warfarin<br>Unknown<br>13 mg<br>Unknown	Miscarriage	No concurrent comorbidities or co-medications were reported.		
**Case 31**<br>Female, unknown,<br>Spontaneous – Physician<br>Serious – other clinical condition<br>Mother-child report	Warfarin<br>Unknown<br>Unknown<br>Unknown	Miscarriage	No concurrent comorbidities or co-medications were reported.		

(describing the mother)					
**Case 32**<br>Female, unknown,<br>Spontaneous – Physician<br>Serious – other clinical condition<br>Mother-child report<br>(describing the mother)	Warfarin<br>Unknown<br>5 mg<br>Unknown	Miscarriage and hemorrhage	No concurrent comorbidities or co-medications were reported.		
**Case 33**<br>Female, unknown,<br>Spontaneous – Physician<br>Serious – other clinical condition<br>Mother-child report<br>(describing the mother)	Warfarin<br>Unknown<br>10 mg<br>Unknown	Miscarriage	No concurrent comorbidities or co-medications were reported.		
**Case 34**<br>Female, 29 years old,<br>Spontaneous – Consumer<br>Unknown<br>Mother-child report<br>(describing the mother)	Warfarin<br>Unknown<br>Unknown<br>Unknown	Miscarriage	<u>Teratogenic drugs</u><br>*Class N*:<br>1) nadroparin calcium	1) Nadroparin – warfarin co-administration can increase the risk of bleeding	Nadroparin's SmPC

**Case 35**<br>Female, 24 years old,<br>Spontaneous – Other healthcare professional<br>Serious – other clinical condition<br>Mother-child report (describing the mother)	Warfarin<br>Unknown<br>1 mg daily<br>Oral	Miscarriage	No concurrent comorbidities or co-medications were reported.		
**Case 36**<br>Female, 35 years old,<br>Spontaneous – Physician<br>Serious – other clinical condition<br>Mother-child report (describing the mother)	Warfarin<br>Unknown<br>Unknown<br>Unknown	Miscarriage	<u>Teratogenic drugs</u><br>*Class N*:<br>1) acetylsalicylic acid<br>2) fondaparinux<br>3) hydroxychloroquine	1) warfarin - acetylsalicylic acid co-administration can increase the risk of bleeding<br>2) warfarin - fondaparinux co-administration can increase the risk of bleeding<br>3) acetylsalicylic acid - fondaparinux co-administration can increase the risk of bleeding	Warfarin's SmPC
**Case 37**<br>Unknown, unknown,<br>Spontaneous – Other healthcare professional<br>Serious – other clinical condition<br>Mother-child report (describing the mother)	Warfarin<br>Unknown<br>Unknown<br>Intravenous (not otherwise specified)	Miscarriage	<u>Teratogenic drugs</u><br>*Class N*:<br>1) ecolizumab<br>*Class B:*<br>1) enoxaparin<br><br>Paroxysmal nocturnal hemoglobinuria	1) enoxaparin – warfarin co-administration can increase the risk of bleeding	Enoxaparin's SmPC [36]

**Case 38**<br>Female, 30 years old,<br>Spontaneous – Unknown<br>Serious – other clinical condition<br>Mother-child report (describing the mother)	Warfarin<br>Unknown<br>Unknown<br>Unknown	Spontaneous abortion and protein urine present	Teratogenic drugs<br>*Class N:*<br>Triamcinolone<br>*Class D*:<br>Captopril<br>Cyclophosphamide<br>Irbesartan<br>*Class C*:<br>Salbutamol<br>Prednisone<br>Furosemide<br>*Class B*:<br>Metolazone	1) concurrent use of angiotensin converting enzyme inhibitors and angiotensin ii receptor blockers may result in increased risk of adverse events (ie, hypotension, syncope, hyperkalemia, changes in renal function, acute renal failure)<br>2) concurrent use of cyclophosphamide and warfarin may result in increased risk for elevated INR and subsequent bleeding<br>3) concurrent use of metolazone and loop diuretics may result in increased risk of electrolyte and fluid imbalance	[37,38]
**Case 39**<br>Female, 35 years old,<br>Spontaneous – Other healthcare professional<br>Serious – other clinical	Warfarin<br>Unknown<br>Unknown<br>Unknown	Spontaneous abortion	Teratogenic drugs<br>*Class N:*<br>Dextropropoxyphene<br>Prochlorperazine	1) concurrent use of enoxaparin and warfarin may result in increased risk of bleeding	Warfarin, imatinib and levofloxacin's SmPCs

condition<br>Mother-child report<br>(describing the mother)			Imatinib<br>Lorazepam<br>Paracetamol<br>*Class C*:<br>Levofloxacin<br>*Class B*:<br>Enoxaparin<br><br>Gastrointestinal stromal tumors	2) concurrent use of acetaminophen and imatinib may result in increased acetaminophen levels<br>3) concurrent use of imatinib and warfarin may result in increased risk of bleeding<br>4) concurrent use of levofloxacin and QT interval prolonging agents may result in increased risk of QT interval prolongation<br>5) concurrent use of levofloxacin and warfarin may result in an increased risk of bleeding	
**Case 40**<br>Female, 23 years old,<br>Spontaneous – Lawyer<br>Serious – caused/prolonged hospitalization<br>Mother-child report<br>(describing the mother)	Warfarin<br>Anticoagulant therapy<br>Unknown<br>Unknown	Spontaneous abortion, Lymphadenitis, migraine, hypotension, lung infiltration, infection, headache, cough, joint injury, asthma, venous insufficiency, intra-uterine death, lower abdominal pain, deep vein	<u>Teratogenic drugs</u><br>*Class N:*<br>Fluticasone<br>Paracetamol<br>*Class X:*<br>Ethinylestradiol + etonogestrel<br>*Class B:*<br>Buspirone<br>Montelukast	1) concurrent use of citalopram and warfarin may result in an increased risk of bleeding	[39]

		thrombosis, and condition aggravated. The mother was exposed to warfarin during the gestational period	*Class C:*<br>Azelastine<br>Metoprolol<br>Salbutamol<br>Citalopram		
**Case 41**<br>Female, 32 years old,<br>Spontaneous – Lawyer<br>Serious – caused/prolonged hospitalization<br>Mother-child report (describing the mother)	Warfarin<br>Anticoagulant therapy<br>Unknown<br>Unknown	Abortion induced, pulmonary embolism, multiple injuries, pain, respiratory failure, caesarean section, hypochromic anemia, cardiac disorder, hypercoagulation, thrombocytopenia, migraine, dizziness, deep vein thrombosis, pulmonary infarction, breast cyst, drug hypersensitivity, ventricular hyperkinesia, gastroesophageal reflux disease, contusion, ecchymosis	Teratogenic drugs<br>*Class N:*<br>Cetirizine<br>Paracetamol<br>*Class B:*<br>Enoxaparin<br>*Class C:*<br>Butalbital<br>Ibuprofen	1) concurrent use of low-molecular-weight heparins and NSAIDs may result in an increased risk of bleeding<br>2) concurrent use of enoxaparin and warfarin may result in increased risk of bleeding<br>3) concurrent use of anticoagulants and NSAIDs may result in increased risk of bleeding	40

**Case 42**<br>Female, 33 years old,<br>Report from study – Physician<br>Serious – caused/prolonged hospitalization<br>Mother-child report (describing the mother)	Warfarin<br>Pulmonary hypertension<br>5 mg daily<br>Oral	Spontaneous abortion, headache	<u>Teratogenic drugs</u><br>*Class N:*<br>Beraprost<br>Sildenafil<br>*Class X:*<br>Ambrisentan<br>*Class D:*<br>Azathioprine<br>*Class C:*<br>Heparin<br>Cyclosporine<br><br>Pulmonary hypertension	1) concurrent use of heparin and warfarin may result in increased risk of bleeding	[35]
**Case 43**<br>Female, 39 years old,<br>Spontaneous – Consumer<br>Serious – caused/prolonged hospitalization<br>Mother-child report (describing the mother)	Warfarin<br>Deep vein thrombosis/Pulmonary embolism<br>Unknown<br>Sublingual	Spontaneous abortion, decreased appetite, deep vein thrombosis, dyspnea, drug withdrawal syndrome, pulmonary embolism, victim of spousal abuse	<u>Teratogenic drugs</u><br>*Class N:*<br>Buprenorphine<br>*Class X:*<br>Drospirenone + ethinylestradiol + levomefolic acid<br>*Class C:*<br>Duloxetine<br>Naloxone<br><br>Maternal age	1) concurrent use of buprenorphine and duloxetine may result in increased risk of serotonin syndrome	Buprenorphine's SmPC

**Case 44**<br>Female, unknown,<br>Spontaneous – Consumer<br>Serious – caused/prolonged hospitalization<br>Mother-child report (describing the mother)	Warfarin<br>Hypercoagulation<br>Unknown<br>Unknown	Abortion induced, gastroesophageal reflux disease, iron deficiency, anemia, pain, thrombocytopenia, hypersensitivity, breast cyst, hypercoagulation, migraine, deep vein thrombosis, ventricular hypokinesia, pulmonary embolism, dyspnea	<u>Teratogenic drugs</u><br>*Class N:*<br>Cetirizine<br>Paracetamol<br>*Class X:*<br>Ethinylestradiol + etonogestrel<br>*Class C:*<br>Ibuprofen<br>Butalbital<br>*Class B:*<br>Enoxaparin	1) concurrent use of low-enoxaparin and ibuprofen may result in an increased risk of bleeding	[40]
**Case 45**<br>Female, unknown,<br>Spontaneous – Lawyer<br>Serious – caused/prolonged hospitalization<br>Mother-child report (describing the mother)	Warfarin<br>Thrombosis<br>Unknown<br>Unknown	Abortion induced, acne, vena cava filter insertion, pulmonary embolism, menstruation delayed, pharyngitis, lymphadenopathy, blood disorder, dermatitis	<u>Teratogenic drugs</u><br>*Class X:*<br>Ethinylestradiol + etonogestrel<br>*Class N:*<br>Mometasone<br>*Class C:*<br>Guaifenesin<br>Heparin<br>Benzoyl peroxide + clindamycin<br>Fluconazole<br>*Class B:*<br>Azithromycin<br>Enoxaparin<br>Metronidazole	1) concurrent use of enoxaparin and heparin may result in increased risk of bleeding<br>2) concurrent use of metronidazole and fluconazole may result in increased risk of QT-interval prolongation and arrhythmias<br>3) concurrent use of fluconazole and warfarin may result in an increased risk of bleeding	[40–42]

				4) concurrent use of metronidazole and warfarin may result in increased risk of bleeding	
**Case 46**<br>Female, 40 years old,<br>Spontaneous – Consumer<br>Serious – other clinical condition<br>Mother-child report (describing the mother)	Warfarin<br>Unknown<br>Unknown<br>Unknown	Spontaneous abortion, hives, dysphagia, coughing, pruritus generalized	<u>Teratogenic drugs</u><br>*Class N:*<br>Nadroparine<br>Acetylsalicylic acid<br>*Class B:*<br>Ticlopidine<br><br>Maternal age	1) concurrent use of listed medicines and warfarin may result in an increased risk of bleeding	[43,44]
**Case 47**<br>Female, 34 years old,<br>Spontaneous – Other healthcare professional<br>Serious – caused/prolonged hospitalization<br>Mother-child report (describing the mother)	Warfarin<br>Mitral valve disease<br>Unknown<br>Unknown	Abortion induced, thrombocytopenia, oral pain, rash pruritic, leukopenia, dysphagia, dermatitis, pyrexia, mouth ulceration, ectopic pregnancy, mucosal inflammation, heart sounds,<br>epidermal necrosis, oropharyngeal pain, hemorrhage	<u>Teratogenic drugs</u><br>*Class X:*<br>Methotrexate<br>*Class N:*<br>Filgrastim<br>*Class D:*<br>Fluconazole<br>*Class B:*<br>Metronidazole<br>Vancomycin<br>Cefepime<br>Acyclovir	1) concurrent use of cefepime and warfarin may result in an increased risk of bleeding<br>2) concurrent use of fluconazole and warfarin may result in an increased risk of bleeding<br>3) concurrent use of methotrexate and warfarin may result in increased risk for elevated INR and subsequent bleeding	[38,42,45,46]

				4) concurrent use of metronidazole and warfarin may result in increased risk of bleeding	
**Case 48**<br>Female, 26 years old,<br>Report from study – Other healthcare professional<br>Serious – Other clinical condition<br>Mother-child report (describing the mother)	Warfarin<br>Deep vein thrombosis<br>7.5 mg daily<br>Oral	Abortion induced	<u>Teratogenic drugs</u><br>*Class N:*<br>Acetylsalicylic acid<br>Lisinopril<br>Carvedilol<br>*Class D*<br>Mycophenolic acid<br>*Class B:*<br>Clopidogrel<br>*Class C:*<br>Prednisone<br>Iron<br>Furosemide	1) concurrent use of warfarin and acetylsalicylic acid may result in increased risk of bleeding<br>2) concurrent use of acetylsalicylic acid and clopidogrel may result in an increased risk of bleeding<br>3) concurrent use of furosemide and acetylsalicylic acid may result in reduced diuretic effectiveness and possible nephrotoxicity	
**Case 49**<br>Female, unknown,<br>Spontaneous – Lawyer<br>Serious – Other clinical condition<br>Mother-child report (describing the mother)	Warfarin<br>Anticoagulant therapy<br>Unknown<br>Oral	Abortion induced	<u>Teratogenic drugs</u><br>*Class X:*<br>Ethinylestradiol + etonogestrel		

**Case 50**<br>Female, unknown,<br>Spontaneous – Lawyer<br>Serious –<br>Caused/Prolonged<br>Hospitalization<br>Mother-child report<br>(describing the mother)	Warfarin<br>Anticoagulant therapy<br>10 mg daily<br>Oral	Spontaneous abortion, pain in extremity, international normalized ratio increased, contusion, deep vein thrombosis, dyspnea, hypercoagulation, heart rate increased, chest pain, pulmonary embolism	<u>Teratogenic drugs</u><br>*Class N:*<br>Bismuth<br>*Class X:*<br>Ethinylestradiol + etonogestrel<br>*Class B:*<br>Enoxaparin	1) concurrent use of enoxaparin and warfarin may result in increased risk of bleeding	Warfarin's SmPC
**Case 51**<br>Male, unknown,<br>Spontaneous – Other healthcare professional<br>Serious – Other clinical condition<br>Child-mother report<br>(describing the child)	Warfarin<br>Unknown<br>Unknown<br>Transplacental	Low birth weight baby and premature baby	<u>Teratogenic drugs</u><br>*Class N:*<br>Acetylsalicylic acid<br>*Class C:*<br>Heparin	1) concurrent use of heparin and warfarin may result in increased risk of bleeding<br>2) concurrent use of acetylsalicylic acid and warfarin may result in increased risk of bleeding<br>3) concurrent use of heparin and acetylsalicylic acid may result in increased risk of bleeding	Warfarin and heparin's SmPCs
**Case 52**<br>Unknown, unknown,<br>Spontaneous – Physician<br>Serious – Congenital anomaly/Birth defect	Warfarin<br>Unknown<br>Unknown<br>Transplacental	Intrauterine growth retardation, fetal malformation, facial dysmorphism, congenital	No concurrent comorbidities or co-medications were reported.		

Child-mother report<br>(describing the child)		great vessel anomaly			
**Case 53**<br>Male, unknown,<br>Spontaneous – Consumer<br>Serious – Congenital anomaly/Birth defect<br>Child-mother report<br>(describing the child)	Warfarin<br>Unknown<br>Unknown<br>Transplacental	Low birth weight baby	No concurrent comorbidities or co-medications were reported.		
**Case 54**<br>Male, unknown,<br>Spontaneous – Other healthcare professional<br>Serious – Congenital anomaly/Birth defect<br>Child-mother report<br>(describing the child)	Warfarin<br>Unknown<br>Unknown<br>Transplacental	Low birth weight baby,<br>APGAR score low,<br>premature baby,<br>chondrodystrophy	<u>Teratogenic drugs</u><br>*Class X:*<br>Lovastatin<br>*Class C:*<br>Citalopram<br>Levetiracetam<br>*Class D:*<br>Topiramate	1) concurrent use of citalopram and topiramate may result in increased citalopram exposure and risk of QT interval prolongation<br>2) concurrent use of citalopram and warfarin may result in an increased risk of bleeding	39

Supplementary Table 5. Two by two tables used to compute reporting odds ratios and 95% confidence intervals in the sensitivity analysis.

Period 2009 – 2017 – cases without alternative causes

Drug	Spontaneous abortion/miscarriage	Other adverse events
**Rivaroxaban**	9	1675
**Other drugs**	8052	1267157
**Reporting Odds ratio (95% Confidence Interval):** 0.85 (0.44-1.63)		
**Interpretation:** Not statistically significant		

Period 2009 – 2017 – all cases

Drug	Spontaneous abortion/miscarriage	Other adverse events
**Rivaroxaban**	23	1661
**Other drugs**	8052	1267157
**Reporting Odds ratio (95% Confidence Interval):** 2.18 (1.44-3.29)		
**Interpretation:** Statistically significant		

Period 2009 – 2017 – cases without alternative causes

Drug	Intrauterine growth retardation/ Low birth weight baby	Other adverse events
**Rivaroxaban**	4	1680
**Other drugs**	286	1274953
**Reporting Odds ratio (95% Confidence Interval):** 10.61 (3.95-28.51)		
**Interpretation:** Statistically significant		

Period 2009 – 2017 – all cases

Drug	Intrauterine growth retardation/ Low birth weight baby	Other adverse events
**Rivaroxaban**	5	1679
**Other drugs**	286	1274953
**Reporting Odds ratio (95% Confidence Interval):** 13.28 (5.48-32.18)		
**Interpretation:** Statistically significant		

Period 2009 – 2017 – all cases

Drug	Spontaneous abortion/miscarriage	Other adverse events
**Apixaban**	6	173
**Other drugs**	8052	1267157
**Reporting Odds ratio (95% Confidence Interval):** 5.46 (2.42-12.32)		
**Interpretation:** Statistically significant		

Period 2009 – 2017 – all cases

Drug	Induced abortion	Other adverse events
**Apixaban**	5	174
**Other drugs**	1922	1273287
**Reporting Odds ratio (95% Confidence Interval):** 19.04 (7.82-46.36)		
**Interpretation:** Statistically significant		
